# From waste to wonder: exploring the hypoglycemic and anti-oxidant properties of corn processing by−products

**DOI:** 10.3389/fchem.2024.1433501

**Published:** 2024-07-18

**Authors:** Xiaoqian Yang, Yuelong Wang, Jingfeng Li, Yuxing Tai, Kunping Yang, Jingwei Lv, Jiaming Sun, Hui Zhang

**Affiliations:** ^1^ Jilin Ginseng Academy, Changchun University of Chinese Medicine, Changchun, China; ^2^ College of Pharmacy, Changchun University of Chinese Medicine, Changchun, China; ^3^ Department of Acupuncture and Tuina, Changchun University of Chinese Medicine, Changchun, China

**Keywords:** corn silk, straw peels, and straw core, hypoglycemic, antioxidant, flavonoids, phenolics, molecular docking, molecular dynamics simulation

## Abstract

**Introduction:** The industrial processing of corn (*Zeamays* L.) generates by-products such as corn silk, straw peels, and straw core, which contribute to adverse environmental impacts. Our study aimed to investigate sustainable approaches for mitigating these effects by evaluating the hypoglycemic potential and mechanisms of ethyl acetate fractions derived from these corn derivatives.

**Methods:** We employed glucose consumption assays, high glucose stress tests, UPLC-QE-Orbitrap-MS analysis, molecular docking, and simulations to assess their components and efficacy. Antioxidant capacities were evaluated using DPPH, FRAP, ABTS, and •OH scavenging assays.

**Results:** Notably, the ethyl acetate fraction extracted from straw peels (SPE) exhibited a high concentration of flavonoids and phenolic compounds along with pronounced hypoglycemic activity and antioxidant capacity. SPE significantly enhanced glucose consumption in insulin-resistant HepG2 cells while protecting HUVECs against damage caused by high glucose levels. Molecular docking analyses confirmed the interaction between active compounds and *α*-glucosidase as well as *α*-amylase, while molecular dynamic simulations indicated stability at their binding sites.

**Discussion:** In conclusion, the hypoglycemic and antioxidative properties observed in corn by-products such as straw peels, corn silk, and straw core can be attributed to the inhibition of *α*-glucosidase and *α*-amylase activities, coupled with their rich phenolic and flavonoid content. These findings highlight the potential of these by-products for applications in healthcare management and their sustainable utilization, demonstrating significant value in the use of agricultural residues.

## 1 Introduction

The metabolic condition of diabetes mellitus (DM) is a complex and chronic disorder that is not transmissible. It presents as impaired glucose tolerance resulting from deficiencies in insulin secretion, insulin function, or both. (Wang and Zhao, 2019). In 2021, the global prevalence of DM was estimated at 500 million, and it is projected to rise by 46%, reaching 783 million by the year 2045 (Saeedi et al., 2019). This surge underscores the significant threat DM poses to global public health. Diabetics face an increased risk of heart disease and stroke by 2-3 times (Emerging Risk Factors et al., 2010), with vascular complications contributing to a staggering mortality rate. One of the hallmark characteristics of diabetes is the augmentation of oxidative stress (OS) under hyperglycemic conditions, which is propelled by glucose autoxidation and the creation of advanced glycation end−products (AGEs), subsequently fostering the production of free radicals and compromising cellular antioxidant defenses (Baiseitova et al., 2023; Qasim et al., 2023). Elevated levels of various lipid peroxidation markers in the plasma and erythrocytes of diabetic patients have been observed, indicating increased oxidative damage throughout the disease progression (Rizvi et al., 2005; Bandeira Sde et al., 2012; Ho et al., 2013). Hyperglycemia further diminishes the antioxidant capacity within tissues by reducing levels of antioxidants such as vitamin E, glutathione, catalase, and superoxide dismutase (SOD), thereby lowering the overall antioxidant defense (Maritim et al., 2003; Contreras-Zentella et al., 2019). Additionally, diabetes alters the structure and functionality of erythrocytes, impacts microcirculation, and promotes the development of complications (Babu and Singh, 2004; Sprague et al., 2006; Zhou et al., 2018). Prolonged hyperglycemia also impairs vascular endothelial cells, fosters the release of inflammatory cytokines, reduces nitric oxide production, leading to vascular dysfunction and atherosclerotic conditions, and elevates the risk of cardiovascular diseases and stroke (Vita and Keaney, 2002; Shi and Vanhoutte, 2017). In the development of complications associated with diabetes, the crucial interplay between OS, the generation of AGEs, and inflammation plays a significant role (Yang et al., 2019). Thus, effective management of OS and improvement of vascular health are essential for the prevention and treatment of diabetes as well as its associated complications.

Currently, the management of type 2 DM (T2DM) predominantly revolves around insulin therapy and a variety of oral antidiabetic agents, including *α-*glucosidase inhibitors, rosiglitazone, sulfonylureas, metformin, and thiazolidinediones (Kim et al., 2006; Lee et al., 2017). However, these pharmacological treatments are often accompanied by notable adverse reactions, including hypoglycemia, gastrointestinal complications, and weight gain (Erpel et al., 2020). Owing to their diverse botanical origins and minimal toxicity, natural products have increasingly garnered interest as potential alternatives in diabetes therapy and natural medicine (Blunt et al., 2013; Barbosa et al., 2014). This shift reflects a growing trend towards exploring more holistic and side−effect−free treatment options for managing T2DM.

Corn, an annual herbaceous plant in the grass family, is one of the top three cereal crops globally, available in abundance throughout the world, especially in China, France, United States and Turkey. Corn silk (Stigma maydis), one of the primary by−products of corn cultivation, is frequently utilized in the traditional medical systems of these regions for treating various ailments (Hasanudin et al., 2012). Recent research indicates that corn silk is rich in polysaccharides, flavonoids, alkaloids, and other chemical components (Xiaolin, 2004; Xiaofei, 2012), which contribute to glucose homeostasis through multiple beneficial effects like anti−hyperglycemic, anti−hyperlipidemic, antioxidative, anti−inflammatory, and immune-enhancing properties (Suzuki et al., 2003; Yang et al., 2014; Xiaoqian, 2019). However, aside from corn seeds and silks, other inedible parts of the plant, such as the straws, are considered waste and have received little attention, with insufficient exploration in scientific research (Hasanudin et al., 2012). As one of the major agricultural countries, China generated an estimated 260 million tons of corn straw in 2020, constituting 33.8% of the total crop straw production for that year (Yang et al., 2023). Large amounts of crop straws remain underutilized and is often disposed of through burning, leading to severe wastage of resources and other environmental issues.

In our previous investigations, we have substantiated the hypoglycemic efficacy of ethyl acetate extracts derived from various components of corn through relevant experimental assays involving *α-*glucosidase and *α-*amylase (Yang et al., 2021a). Therefore, this study reports on the antioxidant, hypoglycemic characteristics, and potential hypoglycemic mechanisms of the ethyl acetate fraction obtained from corn silk (CSE), straw peels (SPE), and straw core (SCE). Initially, we measured the total flavonoid content (TFC) and total phenolic content (TPC) in SPE, SCE, and CSE samples. Subsequently, these extracts were evaluated for their antioxidant capabilities using various assays including 2,2−diphenyl−1−picrylhydrazyl (DPPH), 2,2′−azino−bis (3−ethylbenzothiazoline−6−sulphonic acid) diammonium salt (ABTS), hydroxyl radical (•OH), and ferric reducing antioxidant power (FRAP) methods. The antidiabetic mechanisms of SPE, SCE, and CSE were investigated through *in vitro* hypoglycemic assays, UPLC−QE−Orbitrap−MS analysis, and *in silico* studies. Investigating the composition and biological activity of SPE, SCE, and CSE can provide valuable insights for their potential applications in the fields of food and medicine.

## 2 Materials and methods

### 2.1 Reagents and instrument

Ultra−Performance Liquid Chromatography (UPLC) grade acetonitrile and methanol were obtained from Fisher (Waltham, MA, United States). The water underwent purification using a Milli−Q water purification system (TGI Pure Water Systems, Greenville, SC, United States). Vanquish Duo UPLC-QE-Orbitrap-MS system were obtained from Thermo Fisher Science (CA, United States). DPPH, ABTS, and Folin–Ciocalteu reagent were obtained from Sigma (St. Louis, MO, United States). All other chemicals in this research were of analytical quality. Human umbilical vein endothelial cells (HUVECs) lines were sourced from the Cell Bank of Type Culture Collection Chinese Academy of Sciences (Shanghai, China), while human hepatoma cells (HepG2) were obtained from Procell Life Science &Technology Co., Ltd. (Wuhan, China). Standard compounds such as gallic acid and rutin were provided by the National Institute for the Control of Pharmaceutical and Biological Products (Beijing, China).

### 2.2 Plant materials and extract preparation

Various parts of corn (*Zeamays* L.), including the corn silk, straw peels, and straw core, sourced from Jilin Province in the Northeast region of China, were collected in October 2023. The collected corn by-product samples underwent natural drying at room temperature (20°C–30°C) and voucher specimen (Nos. ZM20231001, ZM20231002 and ZM20231003) have been deposited (for a maximum of 10 weeks) at Composition-activity Relationship of traditional Chinese medicine (TCM) Lab at Jilin Ginseng Academy. Subsequently, the samples were pulverized into fine powder (20 mesh) utilizing an electric grinder (Linda Machinery Co., Ltd, Wenling, Zhejiang, China). The powdered material (50 g) underwent a degreasing process with petroleum ether, followed by extraction with 80% ethanol (1000 mL × 3 times) for 1 h using ultrasonication (110 W). The resultant extracts were filtered using the 5 μm paper and concentrated at 40°C under reduced pressure with a rotary evaporator (Model N-1300, EYELA Corp, Ja-pan). Subsequently, 1.0 g of the concentrated extract was dissolved in 20 mL of purified water, and sequential extractions were conducted using ethyl acetate, yielding SPE, SCE, and CSE. These extracts were then concentrated at 60°C using a rotary evaporator under reduced pressure. Following this, all the dried extracts were precisely weighed and stored at −20°C for further analysis (for a maximum of 10 wk).

### 2.3 Determination of TPC

The modified Folin-Ciocalteu method was used to quantify the TPC (De la Rosa et al., 2011). In brief, 0.5 mL sample solution (0.1 mg/mL) was transferred into a standard colorimetric tube, followed by adding 1 mL of Folin-Ciocalteu reagent. After waiting for 1 min, 2 mL of 10% sodium carbonate solution was added, and the mixture was then diluted to the mark with distilled water. The reaction mixture was then incubated at room temperature in the dark for 2 h. A spectrophotometer (Jena Analytical Instruments Co., Ltd, Thüringen, Germany) was used to record the absorbance at a wavelength of 765 nm. To quantify TPC values as milligrams of gallic acid equivalents (GAE) per gram of extract (mg · GAE/g), we created a standard calibration curve using gallic acid. This procedure was replicated in three independent experiments.

### 2.4 Determination of TFC

The method described by (Sdayria et al., 2018) was used for the determination of TFC, with slight modifications. A 0.5 mL aliquot of the sample solution (0.1 mg/mL) was introduced into a standard colorimetric tube with a volume adjustment to 5 mL using 70% ethanol. Subsequently, 0.3 mL of 5% NaNO_2_ was added separately to the mixture, followed by the addition of 0.3 mL Al (NO_3_)_3_ (10%) after 6 min and then 2 mL of 4% NaOH after an additional minute. The volume was adjusted accordingly using 70% ethanol and the samples were incubated at room temperature for a duration of 15 min. Absorbance readings were taken at a wavelength of 765 nm utilizing a spectrophotometer (Jena Analytical Instruments). To establish a standard calibration curve, quercetin was employed as reference compound. The TFC was quantified in terms of milligrams of rutin equivalents (RE) per gram of extract (mg RE/g). This procedure was replicated in three independent experiments.

### 2.5 Determination of oxidoreductive activities

#### 2.5.1 DPPH assay

The Nunes method (Nunes et al., 2017), with minor modifications, was used to measure the scavenging activity of the assay extracts against DPPH. An amount of 1 mL of SPE, SCE and CSE (0-1 mg/mL) were combined with 1 mL of 0.2 mM DPPH ethanol solution. The mixture was shaken and left to react in darkness for 30 min. The absorbance (A) was recorded at 517 nm using spectrophotometer (Jena Analytical Instruments). To obtain $A_{0}$ , 1 mL of methanol was used instead of the DPPH solution, and for $A_{1}$ , 1 mL of methanol replaced the sample solution. Butylated hydroxytoluene (BHT) served as the positive control. The DPPH scavenging activity was determined using the following formula:

$$\text{DPPH }\text{scavenging }\text{activity }\%=\frac{1-\left(A-A_{0}\right)}{A_{1}}\times 100\%$$

#### 2.5.2 ABTS assay

The method of Lin (Erhabor et al., 2021), subject to minor modifications, was used to measure the scavenging activity of the assay extracts against ABTS. An amount of 1 mL of SPE, SCE and CSE extracts (0-1 mg/mL) were each mixed with 1 mL of ABTS working solution. The mixture was then incubated at 37°C ± 2°C for 30 min. The absorbance value was measured using spectrophotometer (Jena Analytical Instruments) at 734 nm, denoted as A. For the absorbance $A_{0}$ , 1 mL of 70% ethanol was used instead of the ABTS solution. Additionally, $A_{1}$ was determined by mixing 1 mL of ABTS solution with 20 µL of 70% ethanol. BHT served as a positive control. The ABTS scavenging activity was calculated using the following formula:

$$\text{ABTS }\text{scavenging }\text{activity }\%=\frac{1-\left(A-A_{0}\right)}{A_{1}}\times 100\%$$

#### 2.5.3 •OH assay

The method of Li (Li et al., 2022), with minor modifications, was used to measure the scavenging •OH activity of the tested extracts. An amount of 1 mL of SPE, SCE and CSE (0-1 mg/mL) extracts were added to 200 µL of 9 mmol/L FeSO_4_, 200 µL of 9 mmol/L salicylic acid, and 200 µL H_2_O_2_, respectively. After the mixture was incubated for 30 min at 37°C ± 2°C. The absorbance value was measured using spectrophotometer (Jena Analytical Instruments) at 510 nm, marked as A. The absorbance value A_1_ was treated with 50 µL ethanol replaced by sample solution. And the absorbance value A_0_ was treated with 200 µL salicylic acid with 200 µL methanol solution, BHT was used as a positive control. The scavenge ⋅OH was calculated according to the formula:

$$•\text{OH }\text{scavenging }\text{activity }\%=\frac{1-\left(A-A_{0}\right)}{A_{1}}\times 100\%$$

#### 2.5.4 FRAP assay

The method of Masood (Masood et al., 2021), with minor modifications, was used to measure the scavenging activity of the assay extracts using the FRAP method. An amount of 15 µL of SPE, SCE and CSE (0-1 mg/mL) extracts were added to 150 µL of FRAP working solution, and the mixture was incubated for 5 min at 37°C ± 2°C. BHT was used as a positive control. The absorbance value was measured using spectrophotometer (Jena Analytical Instruments) at 592 nm, The ferrous sulfate (100–1000 µM) was applied to make a standard curve, and expressed as mg iron (II) sulfate equivalent ferrous sulfate for 1 g of raw material used.

### 2.6 Cell culture and viability assay

HepG2 and HUVECs lines were maintained routinely minimum essential medium (MEM) and Dulbecco’s modified eagle medium (DMEM) medium supplemented with 10% (v/v) fetal bovine serum, 1% (v/v) penicillin–streptomycin solution (100x) at 37°C in an atmosphere of 5% CO_2_ in a humidified incubator. Cells (5 × 10^3^ /mL) were seeded in a 96−well plate and treated with 0.125, 0.25, 0.50 mg/mL of SPE, SCE and CSE. After incubating the cells for 24 h, 10 µL of Cell Counting Kit-8 (CCK-8) solution was added to each well and then incubated for an additional 2 h at 37°C. The absorbance of each well was subsequently measured at 450 nm wavelength.

#### 2.6.1 Anti−insulin resistance activity

In order to establish an *in vitro* model of insulin resistance (IR), HepG2 cells were treated with insulin. SPE, SCE, and CSE extracts (0.125, 0.25, 0.5 mg/mL) and metformin (positive control) were added and incubated for another 24 h. Glucose levels in the supernatants were measured using a glucose assay kit (Nanjing Jiancheng Bioengineering Institute, Nanjing, China). Cells were processed for Glucokinase (GK) and Glucose-6-phosphatase (G-6-P) analysis using ELISA kits after freeze-thaw cycles and sonication. For detailed experimental procedures, see the Supplementary Material.

#### 2.6.2 HUVECs damage induced by high glucose

Endothelial dysfunction was induced using 40 mmol/L high glucose for 24 h. SPE, SCE, and CSE extracts (0.125, 0.25, and 0.5 mg/mL) were then added and incubated for another 24 h. Rosiglitazone was used as a positive control. Cells were washed with PBS, digested with trypsin, centrifuged, and stored at −80°C after freezing in liquid nitrogen. After five freeze-thaw cycles, cellular contents were released by ultrasonic disruption. The supernatant was collected after centrifugation to measure t-PA, ET-1, NO, and PAI-1 levels with ELISA kits. For detailed experimental procedures, see the Supplementary Material.

### 2.7 UPLC−QE−Orbitrap−MS analysis

The analysis of SPE, SCE and CSE were carried out using a Vanquish Duo UPLC System (Waltham, MA, United States) and Q Exactive Focus mass spectrometer (Thermo Fisher Science, San Jose, CA, United States). A Hyersil Gold TM chromatographic column (2.1 mm × 50 mm, 1.9 µm) was employed for chromatograph separation at a temperature of 25°C. The injection volume was 5 µL with a flow rate of 0.4 mL/min. The mobile phase comprising 0.1% formic acid aqueous solution (A) and acetonitrile (B). The gradient elution program was as follows: 5% B for 0–4 min, 5%–15% B for 4–8 min; 15%–29% B for 8–16 min, 29%–90% B for 16–25 min; 90%–95% B for 25–35 min, 95% B for 35–40 min. The electron spray ionization (ESI) technique was employed for the ion source, with the mass spectrometer (MS) operating in negative mode. The scanning mode used was full scan/ddMS2, specifically designed for negative ion scanning within a range of 100–1,500 Da. A capillary temperature of 250°C was maintained throughout the analysis. In negative mode, a spray voltage of 3500 V was applied, ensuring full MS resolution at 70,000 and ddMS2 resolution at 35,000. The collision energy utilized in normalized collisional energy (NCE) mode was 30/35/40 eV.

### 2.8 Molecular docking

To begin, protein and ligand files were obtained from the RCSB Protein Data Bank (https://www.rcsb.org/) in “PDB” and “SDF” formats, respectively. Identify the matching coordinates for the protein and the ligand. The protein files were converted to “PDBQT” format to ensure compatibility with molecular docking software. Similarly, the compounds identified in SPE, SCE, and CSE, downloaded in “SDF” format, were converted into “PDBQT” ligand format. Molecular docking studies were conducted using AutoDock Vina 1.2.2 (Center for Computational Structural Biology, United States) to evaluate the binding affinities between the target proteins and the ligands. Visualize these outcomes using PyMOL 2.0 software (DeLano Scientific LLC, United States), showcasing hydrogen bonding interactions and amino acid residues involved in the binding process.

### 2.9 Molecular dynamics simulation

The initial molecular dynamics simulation conformation was derived from the outcomes of molecular docking. Gromacs (released 12 November 2018), a dynamic simulations software, was utilized with the Charmm 36 force field and TIP3P water model. A water box was established and sodium ions were incorporated to maintain system balance. To optimize the molecular configuration, an energy minimization process was conducted on the entire system. Afterwards, the system was subjected to equilibration using both the canonical ensemble and isothermal-isobaric ensemble at normal temperature and pressure for a period of 100 ns, with the aim of conducting molecular dynamics simulation. The simulation trajectory underwent further analysis employing root mean square fluctuation (RMSF), root mean square deviation (RMSD), and the radius of gyration of the protein backbone to assess dynamic stability and conformational changes.

### 2.10 Statistical analysis

Statistical analyses were performed using SPSS v22.0 (IBM, Armonk, NY, United States) and GraphPad Prism 9.0 (GraphPad Software, Boston, MA, United States). Analysis of variance (ANOVA) with 95% confidence intervals was utilized to identify significant differences among the mean values.

## 3 Results

### 3.1 Determination of total phenolics and flavonoids content

Remarkable differences were evident in the TPC among SPE, SCE, and CSE, as detailed in Table 1. The TPC ranged from 93.6 to 215.1 mg GAE/g of dry extract. SPE stood out with the highest TPC, reaching 215.1 ± 1.90 mg/g, followed by SCE with 160.9 ± 0.62 mg/g, and CSE at 93.6 ± 0.94 mg/g. When examining TFC, the values spanned from 80.0 to 182.3 mg RE/g of dry extract. SPE again led the pack with a TFC of 182.3 ± 0.16 mg/g, while CSE and SCE followed with 80.0 ± 0.31 mg/g and 58.0 ± 0.21 mg/g, respectively. It’s particularly noteworthy that SPE not only dominated in TPC but also in TFC, establishing it as the most phenolic and flavonoid-rich extract among the samples analyzed.

**TABLE 1:** Total phenolic and flavonoid contents of SPE, SCE, and CSE.

Samples	TPC (mg · GAE/g extract)	TFC (mg · RE/g extract)
SPE	215.1 ± 1.90^a^	182.3 ± 0.16^a^
SCE	160.9 ± 0.62^b^	58.0 ± 0.21^c^
CSE	93.6 ± 0.94^c^	80.0 ± 0.31^b^

^a−c^ Columns with different superscripts indicate a significant difference (*p* < 0.05).

SPE (straw peels of ethyl acetate fraction), SCE (straw core of ethyl acetate fraction) and CSE (corn silk of ethyl acetate fraction).

TPC: Total phenolic content. TFC: Total flavonoid content.

GAE: Gallic acid equivalent. QE: Rutin equivalent.

Values are the mean ± standard deviation of three independent experiments.

### 3.2 Antioxidant activity *in vitro*

To comprehensively evaluate the antioxidant activity of SPE, SCE, and CSE, we employed multiple assays, including DPPH, ABTS, •OH scavenging capacity, and FRAP. This multifaceted approach was necessary due to the distinct mechanisms of action associated with each antioxidant assay. IC_50_ value is a widely used parameter for measuring antioxidant activity. A lower IC_50_ indicates higher antioxidant activity (Table 2).

**TABLE 2:** Antioxidant activity of SPE, SCE and CSE determined with DPPH, ABTS, •OH and FRAP.

Samples	DPPH IC_50_ (mg/mL)	ABTS IC_50_ (mg/mL)	•OH IC_50_ (mg/mL)	FRAP IC_50_ (mg/mL)
SPE	0.24 ± 0.00^b^	0.05 ± 0.00^a^	0.40 ± 0.01^b^	0.63 ± 0.02^b^
SCE	0.42 ± 0.02^c^	0.11 ± 0.01^b^	0.62 ± 0.03^d^	0.76 ± 0.01^c^
CSE	0.61 ± 0.02^d^	0.13 ± 0.01^c^	0.56 ± 0.00^c^	1.06 ± 0.05^d^
BHT	0.15 ± 0.00^a^	0.14 ± 0.02^d^	0.28 ± 0.02^a^	0.43 ± 0.01^a^

^a−d^ Columns with different superscripts indicate a significant difference (*p*< 0.05).

SPE (straw peels of ethyl acetate fraction), SCE (straw core of ethyl acetate fraction) and CSE (corn silk of ethyl acetate fraction).

Used as a standard antioxidant.

DPPH: 2,2−Diphenyl−1−picrylhydrazyl. ABTS: 2,2′−Azino−bis (3−ethylbenzothiazoline−6−sulphonic acid) diammonium salt. FRAP: Ferric−reducing antioxidant power. •OH: Hydroxyl radicals.

IC_50_: Concentration required to scavenge 50% of the radicals present in the test solution. BHT: butylated hydroxytoluene.

Values are the mean ± standard deviation of three independent experiments.

#### 3.2.1 DPPH assay

DPPH radicals serve as a common metric for assessing the free radical scavenging potential of natural compounds (Zhao et al., 2022). Table 2 illustrates the free radical scavenging activities of SPE, SCE, and CSE, benchmarked against BHT. The SPE (IC_50_, 0.24 ± 0.00 mg/mL) exhibited high scavenging activity, as did BHT (IC_50_, 0.15 ± 0.00 mg/mL). However, SCE (IC_50_, 0.42 ± 0.02 mg/mL) and CSE (IC_50_, 0.61 ± 0.02 mg/mL) exhibited weak scavenging activity. In general, the SPE possesses superior antioxidant properties compared to SCE and CSE.

#### 3.2.2 ABTS assay

The ABTS radical scavenging assay is frequently employed to assess the effectiveness of a sample in neutralizing the ABTS radical (Fan et al., 2020; Wang et al., 2020). The scavenging ability of SPE, SCE, and CSE against the ABTS radical exhibited an enhancement with rising mass concentrations, demonstrating a positive linear correlation. Table 2 reveals that SPE demonstrated robust scavenging activity at a low concentration with an IC_50_ value of 0.05 ± 0.00 mg/mL, which is 2-3 times more effective than the positive controls. Followed by SCE (IC_50_, 0.11 ± 0.01 mg/mL) and CSE (IC_50_, 0.13 ± 0.01 mg/mL) showed strong scavenging activity.

#### 3.2.3 Hydroxyl radicals assay

Table 2 showed SPE displayed moderate hydroxyl radical scavenging activity with the IC_50_ value as 0.40 ± 0.01 mg/mL. The IC_50_ values indicate that the hydroxyl radical scavenging activity of the ethyl acetate fraction from corn derivatives ranks as SPE > CSE > SCE. SPE (IC_50_, 0.40 ± 0.01 mg/mL) and BHT (IC_50_, 0.28 ± 0.02 mg/mL) exhibited high efficiency as hydroxyl radical scavengers, while SCE demonstrated the lowest scavenging activity with an IC_50_ of 0.62 ± 0.03 mg/mL.

#### 3.2.4 FRAP assay

Antioxidants in the samples can reduce ferric tripyridyltriazine (Fe^3+^ TPTZ) to ferrous tripyridyltriazine (Fe^2+^ TPTZ), serving as a measure of their antioxidative capability (Masood et al., 2021). The tested results were consistent with the Hydroxyl radical assay, SPE, SCE, and CSE showed ferric reducing antioxidation power (Table 2), SPE possessed stronger ferric reducing antioxidation power (IC_50_, 0.40 ± 0.01 mg/mL), while SCE, and CSE showed lower ferric reducing antioxidation power than BHT (IC_50_, 0.28 ± 0.02 mg/mL).

### 3.3 Anti−insulin resistance and cell viability assays

**FIGURE 1:**
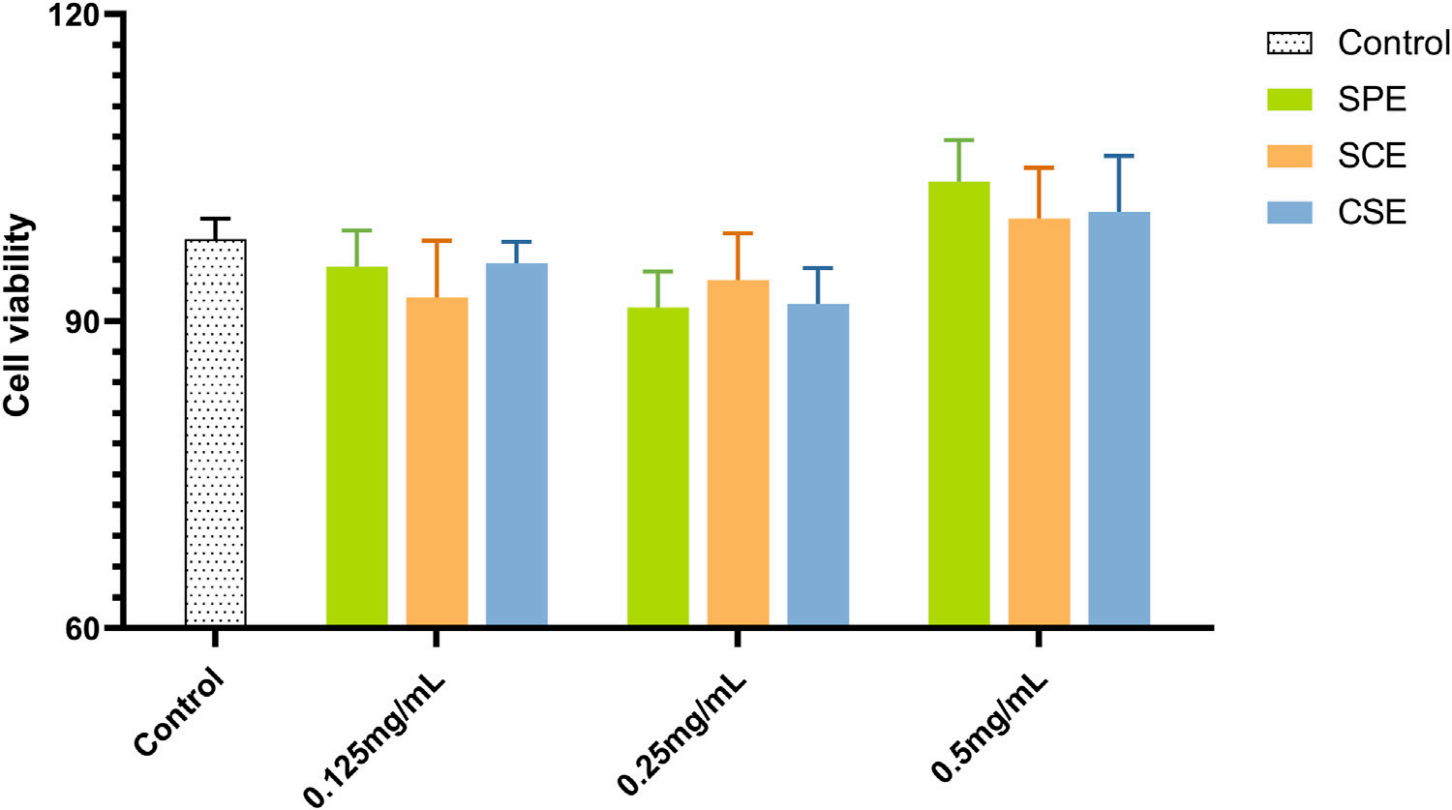
Cell viability assays results of SPE, SCE, and CSE. SPE (straw peels of ethyl acetate fraction), SCE (straw core of ethyl acetate fraction) and CSE (corn silk of ethyl acetate fraction).

**FIGURE 2:**
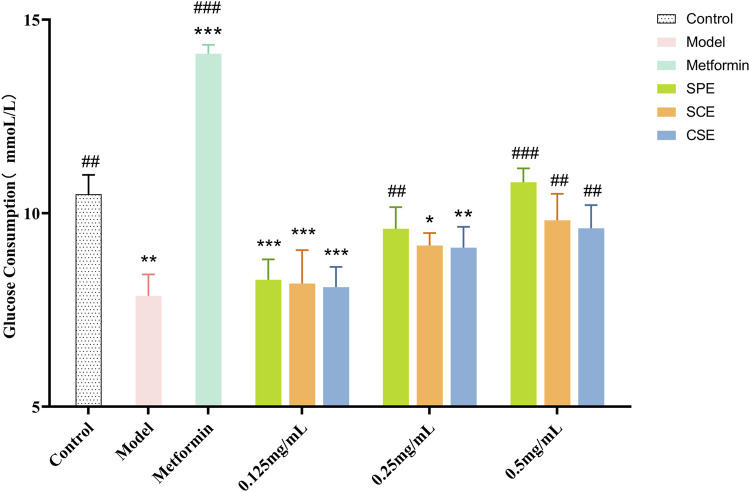
Glucose consumption of SPE, SCE and CSE. SPE (straw peels of ethyl acetate fraction), SCE (straw core of ethyl acetate fraction) and CSE (corn silk of ethyl acetate fraction). Significantly different from the control group at *p < 0.05, **p < 0.01 and ***p < 0.001. Significantly different from the Model group at #*p* < 0.05, ##*p* < 0.01 and ###*p* < 0.001. Values are expressed as the mean ± standard error of the mean (n = 3).

IR-HepG2 cells were used to measure glucose consumption and cell viability across different groups (model, positive control, SPE, SCE, and CSE), with metformin as the positive control. As shown in Figure 1 (Supplementary Table S1), the SPE, SCE, and CSE groups had minor inhibitory effects on HepG2 viability. Additionally, 0.5 mg/mL SPE and SCE slightly promoted HepG2 viability. Other groups showed no significant impact on cell viability. When the HepG2 was treated with IR, there was an obvious decrease, from 10.49 mM to 7.87 mM, in the content of glucose consumption compare to the control group (Figure 2; Supplementary Table S2). The above results demonstrated that a model of IR in HepG2 was successfully built. According to Figure 2, the glucose concentrations in the media of SPE groups at doses of 0.125, 0.25, and 0.5 mg/mL were 8.28 ± 0.52, 9.6 ± 0.55, and 10.8 ± 0.36 mmol/L, respectively, after 24 h of treatment. Notably, concentrations of 0.25 and 0.5 mg/mL of SPE, SCE, and CSE significantly enhanced glucose consumption in IR-HepG2 cells, with the differences being statistically significant. A concentration-dependent effect in promoting glucose consumption was observed with SPE, SCE, and CSE. In conclusion, SPE demonstrated the most potent protective effect on glucose consumption, with its impact at 0.5 mg/mL surpassing even that observed in the normal control groups. Additionally, SPE significantly enhanced glucose consumption in IR-HepG2 cells without adversely affecting cell viability.

### 3.4 Intracellular enzyme activity analysis

IR is the primary cause for the early onset of diabetes, with the liver serving as the primary site of insulin action and a major contributor to IR. This resistance leads to alterations in intracellular enzyme activity, particularly affecting GK, which plays a crucial role in catalyzing glucose phosphorylation. Consequently, once glucose enters the cell via phosphorylation, its ability to freely exit through the cell membrane is hindered. GK represents the initial rate−limiting enzyme in glucose metabolism, and its activity is significantly diminished in IR cells. G−6−P, also known as Glucose−6−phosphatase, is an enzyme that plays a crucial role in regulating blood glucose levels by catalyzing the hydrolysis of glucose−6−phosphate, resulting in the release of glucose into the bloodstream. This enzymatic process serves as the final step in both gluconeogenesis and glycogen decomposition. Notably, in cells exhibiting insulin resistance, there is a notable elevation in G−6−P activity.

**FIGURE 3:**
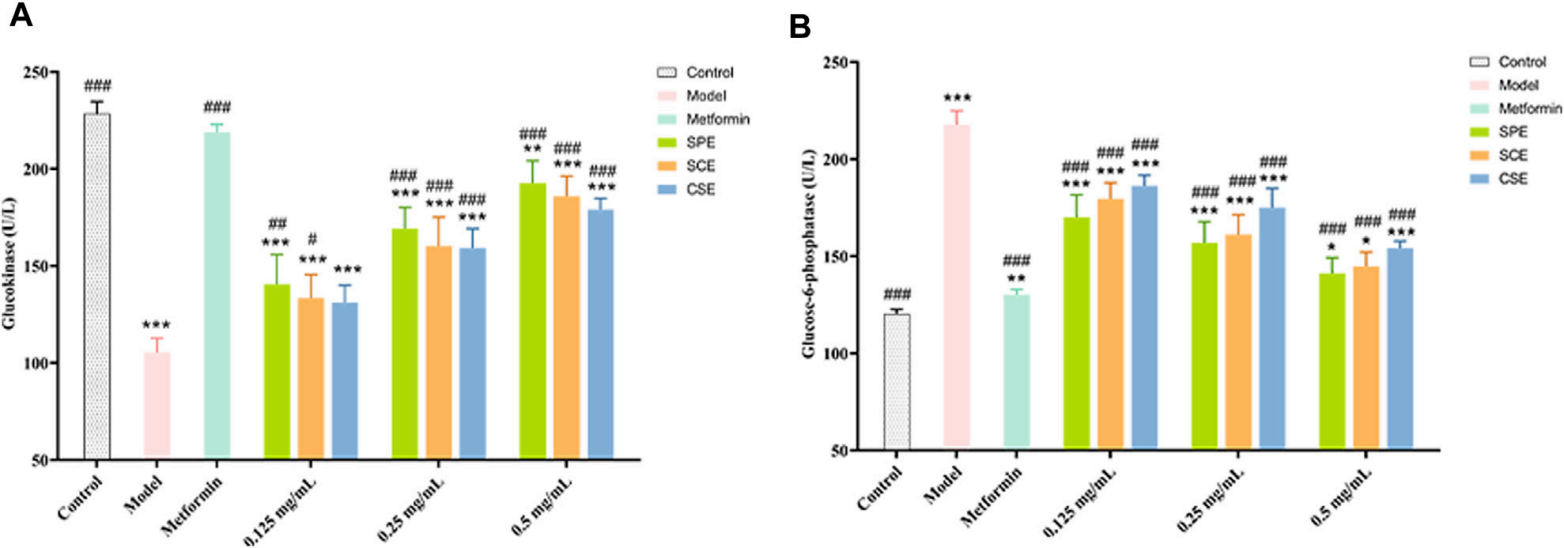
The outcomes of treatment with SPE, SCE, and CSE on the **(A)** GK (Glucokinase) and **(B)** G-6-P (Glucose−6−phosphatase) activity in IR−HepG2. SPE (straw peels of ethyl acetate fraction), SCE (straw core of ethyl acetate fraction) and CSE (corn silk of ethyl acetate fraction). Significantly different from the control group at *p < 0.05, **p < 0.01 and ***p < 0.001 Significantly different from the Model group at #*p* < 0.05, ##*p* < 0.01 and ###*p* < 0.001. Values are expressed as the mean ± standard error of the mean (n = 3).

Glucose consumption as a conventional method for directly assessing glucose metabolism in HepG2 within an IR model. Metformin was chosen as a positive control drug. As depicted in Figure 1, the model group exhibited a substantial inhibition in glucose consumption. In order to investigate the therapeutic impact of SPE, SCE and CSE on IR, the activities of GK and G−6−P were assessed. As depicted in Figure 3A (Supplementary Table S3), the GK activity in the model group was considerably suppressed, whereas the application of the sample led to a substantial dose−dependent increase in GK activity. Particularly, when the concentrations of SPE and SCE reached 0.125 mg/mL, they exhibited significant deviations from the model group. Furthermore, based on Figure 3B (Supplementary Table S4), in the model group, the activity of G-6-P exhibited a significant increase. Conversely, the application of the sample led to a significant decrease in G-6-P activity, demonstrating a dose-dependent relationship The assessment of these two indicators yielded intriguing findings. Notably, SPE exhibited a stronger therapeutic effect on insulin resistance compared to SCE and CSE, and in certain aspects, its efficacy was comparable to that of metformin, thus emphasizing its potential in hypoglycemic interventions.

### 3.5 HUVECs damage induced by high glucose

**FIGURE 4:**
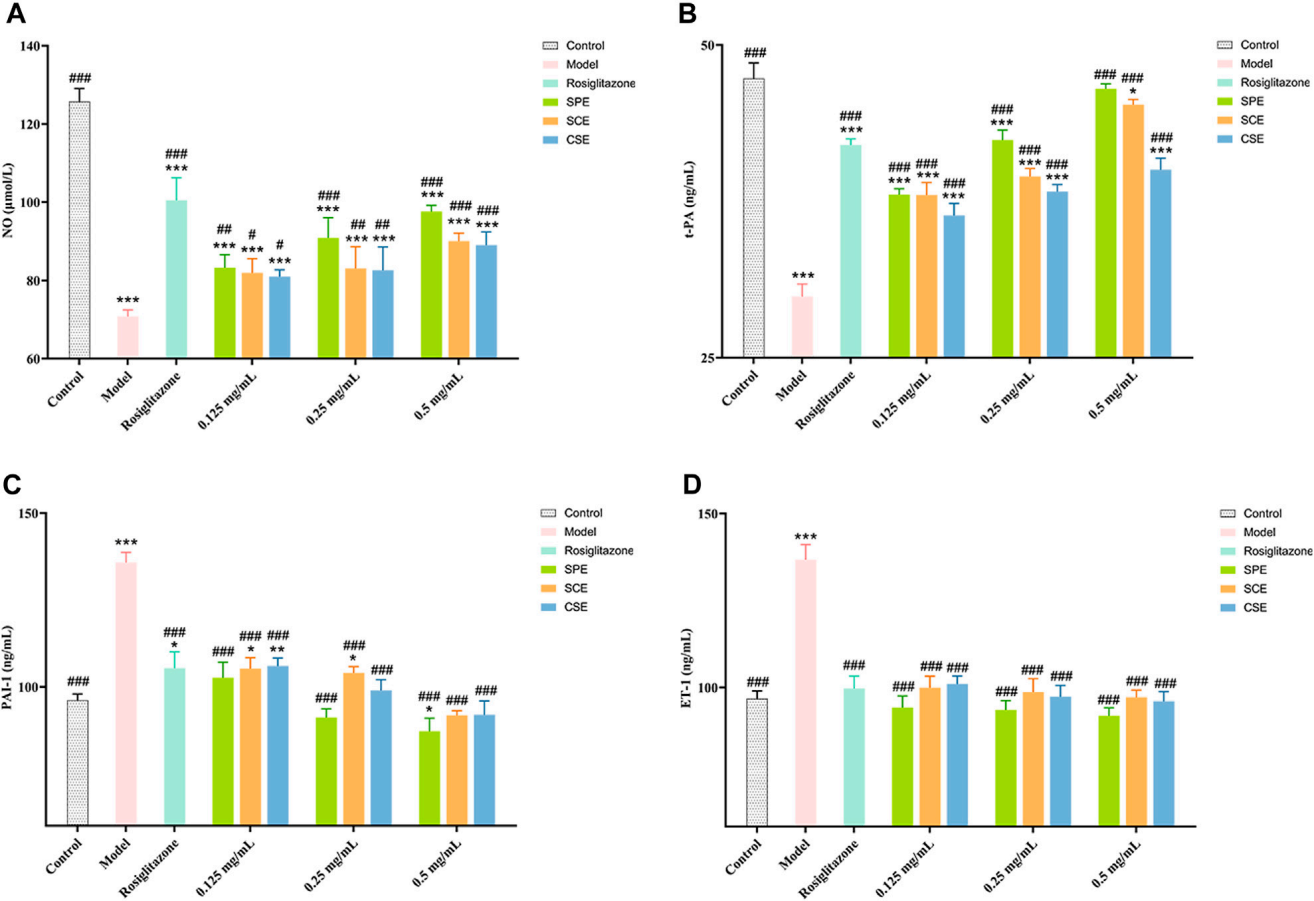
The outcomes of treatment with SPE, SCE, and CSE on the **(A)** NO (nitric oxide), **(B)** ET−1 (endothelin−1), **(C)** PAI−1 (plasminogen activator inhibitor 1) and **(D)** t−PA (tissue−type plasminogen ac-tivator) activity in HUVECs. SPE (straw peels of ethyl acetate fraction), SCE (straw core of ethyl acetate fraction) and CSE (corn silk of ethyl acetate fraction). Significantly different from the control group at *p < 0.05, **p < 0.01 and ***p < 0.001 Significantly different from the Model group at #*p* < 0.05, ##*p* < 0.01 and ###*p* < 0.001. Values are expressed as the mean ± standard error of the mean (n = 3).

In this study, we conducted an assessment of the SPE, SCE, and CSE protective effect on HUVEC injury induced by diabetes, with rosiglitazone serving as a positive control. In Figure 4 (Supplementary Tables S5–S8), our experimental findings demonstrate that under high glucose conditions, the levels of crucial biomarkers NO and t−PA exhibit a significant decrease, whereas the levels of PAI−1 and ET−1 experience a significant increase. These observations align with the vascular injury and dysfunction associated with diabetes. Following treatment with positive drugs and extracts (SPE, SCE, and CSE), there was a noteworthy enhancement in the levels of these biomarkers within the impaired cells, indicating a dose−dependent association. As shown in Figures 4A, B, the activities of NO and t−PA were significantly decreased in the Model compared with the Control group (*p* < 0.01). The activities of NO and t−PA were significantly increased in the SPE, SCE, and CSE groups compared with the Model (*p* < 0.01 or *p* < 0.05). As depicted in Figures 4C, D, at 0.5 mg/mL, the inhibitory effects of SPE, SCE, and CSE on PAI-1 and ET-1 surpassed those of rosiglitazone, with SPE exhibiting the most pronounced effect. It is important to note that the use of rosiglitazone at high concentrations may excessively inhibit PAI-1 and ET-1 expression, potentially compromising vascular function. In contrast, low-dose extracts (SPE, SCE, and CSE) effectively restore PAI-1 and ET-1 expression to nearly normal levels, thereby potentially mitigating the adverse effects associated with high-dose rosiglitazone. As illustrated in Figure 4B, at concentrations ranging from 0.125 to 0.5 mg/mL, the SPE, SCE, and CSE demonstrates a more pronounced enhancing effect on t−PA compared to rosiglitazone, highlighting its potential in enhancing the fibrinolytic system. This implies that the SPE, SCE, and CSE potentially possesses a safeguarding influence on diabetes−induced vascular injury through the regulation of these pivotal molecules’ expression. In summary, the aforementioned findings establish a robust scientific foundation for the utilization of SPE, SCE and CSE in the mitigation and management of cardiovascular and cerebrovascular complications associated with diabetes. This intervention not only ameliorates diabetes−induced vascular injury, but also enhances vascular functionality through the modulation of pivotal biomarker expression. Consequently, it is anticipated that this intervention will serve as an efficacious adjunctive therapy for cardiovascular and cerebrovascular complications.

### 3.6 Chemical components

It has been documented that corn derivatives possesses a diverse array of complex heterogeneous components, including phenolics, flavonoids, lipids, proteins, carbohydrates, and polysaccharides (Hamzah et al., 2021; Zhang et al., 2021; Hossain and Jayadeep, 2022). Its chemical composition, however, has not been elucidated in many studies. In this research, Compound Discoverer 3.0 (Thermo Fisher Science) was employed to analyze the extracted molecular ion chromatographic peaks and isotope peaks using the raw data obtained from UPLC−QE−Orbitrap−MS. Subsequently, the results were cross-referenced with online databases (mz Cloud and mz Vault) and relevant literature, leading to the identification of 61 compounds present in the extracts of SPE, SCE, and CSE (refer to Supplementary Table S9).

### 3.7 Molecular docking

**FIGURE 5:**
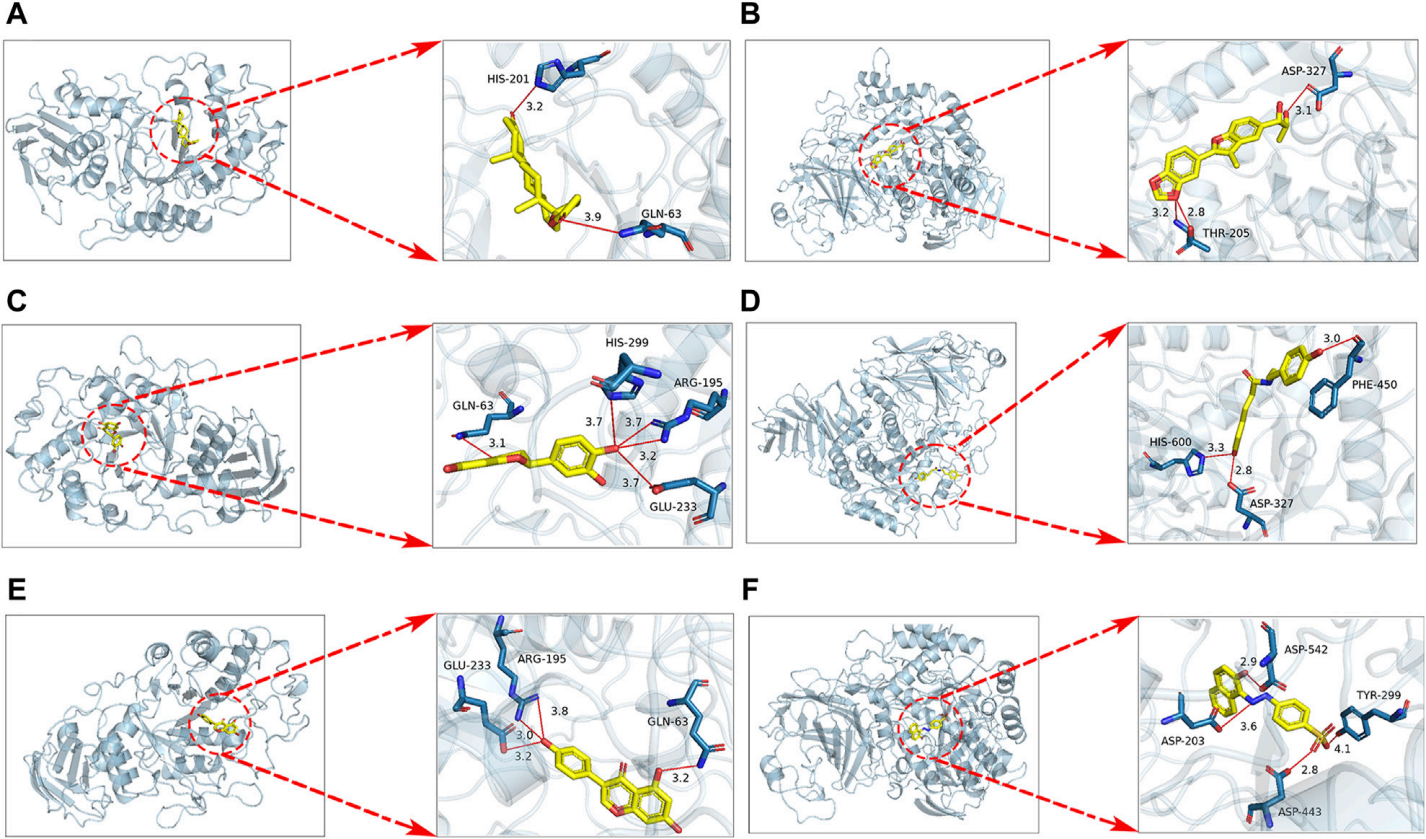
Docking results for SPE, SCE, MSE system with α-amylase, *α*-glucosidase molecules are shown. **(A)** Docking of canrenone with *α*-amylase. **(B)** Docking of 1−[2−(1,3−benzodioxol−5−yl) −3−methyl−1−benzofuran−5−yl] propane−1,2−diol (CHEBI:190940) with *α*-glucosidase. **(C)** Docking of erodictyol with *α*-amylase. **(D)** Docking of paprazine with *α*-glucosidase. **(E)** Docking of genistein with *α*-amylase. **(F)** Docking of mandarin G with *α*-glucosidase.

The results of molecular docking analysis indicate that in the SPE system, the compound canrenone and 1−[2−(1,3−benzodioxol−5−yl) −3−methyl−1−benzofuran−5−yl] propane−1,2−diol (CHEBI:190,940), exhibits the strongest binding affinity towards α-amylase and α-glucosidase, with binding energies of −10.2 and −7.8 kcal/mol, respectively. Similarly, in the SCE system, the compounds erodictyol and paprazine demonstrate the lowest binding energies towards *α*-amylase and *α*-glucosidase, with values of −9.2 and −7.8 kcal/mol, respectively. Lastly, in the CSE system, the compounds genistein and mandarin G exhibit the lowest binding energies towards *α*-amylase and *α*-glucosidase, with values of −8.9 and −7.7 kcal/mol, respectively. A binding energy below −4.0 kcal/mol indicates favorable molecule-protein binding. In this instance, all candidate compounds recorded binding energies below −4.0 kcal/mol, indicating robust interactions with *α*-amylase and *α*-glucosidase. Visualization of these interactions was conducted using PyMOL 2.0 software, as depicted in Figure 5.

### 3.8 Dynamics simulation

Molecular dynamics simulations were utilized to confirm the stability of compound-target protein interactions after docking. In this investigation, the CHARMM force field was applied, and a 200 ns simulation was conducted to assess the structural integrity of the complex. The trajectory underwent analysis for RMSD, RMSF, and protein gyration radius (RG). RMSD is a critical metric for evaluating system stability. Throughout the simulation, a consistently low RMSD value suggested a stable complex with strong ligand-receptor binding.

#### 3.8.1 Root mean square deviation analysis

**FIGURE 6:**
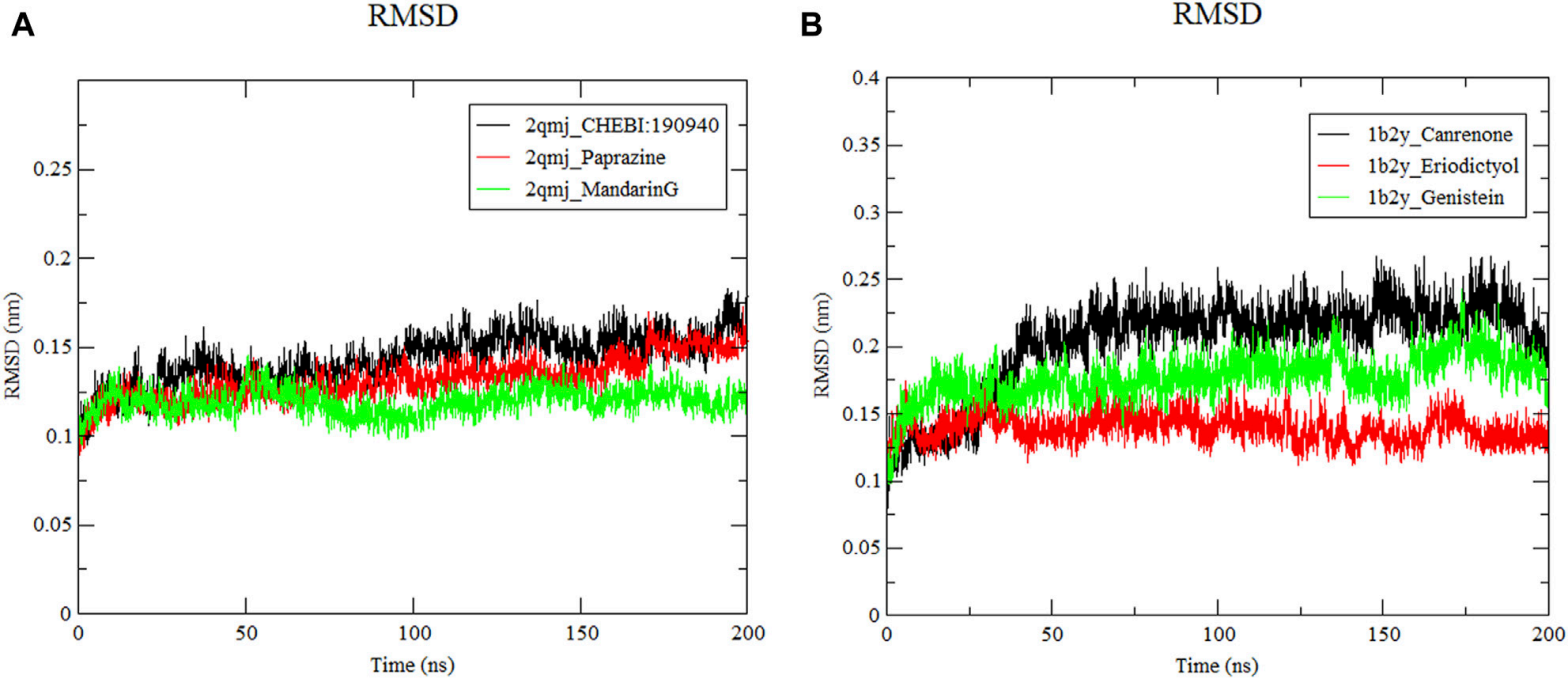
Root mean square deviation of ligand−protein systems. **(A)** 2qmj is *α*-glucosidase, Ligand is CHEBI:190940, paprazine and mandarinG **(B)** 1b2y is *α*-amylase, Ligand is canrenone, eriodictyol and genistein.

The RMSD quantifies the average displacement deviation of a complex relative to a reference over a specific simulation duration, with a lower value indicating greater stability of the system (Ebenezer et al., 2023). As illustrated in Figure 6, the receptor protein across all docking systems exhibited notable stability, maintaining an RMSD value below 0.2 nm. Among these *α*-glucosidase−CHEBI:190940, *α*-glucosidase−paprazine, *α*-glucosidase−mandarinG and *α*-amylase−canrenone, α-amylase−eriodictyol, α-amylase−genistein showed minimal movement with stable RMSF values after 50 ns. The RMSD values of *α*-glucosidase−CHEBI:190940, *α*-glucosidase−paprazine, *α*-glucosidase−mandarinG and *α*-amylase−canrenone, *α*-amylase−eriodictyol, *α*-amylase−genistein fluctuated at 0.1 nm and 0.3 nm, respectively, during the 200 ns simulation. However, the ligands remained in the active pockets of α-glucosidase and α-amylase during the simulation, indicating stable complexes.

#### 3.8.2 Root mean square fluctuation analysis

**FIGURE 7:**
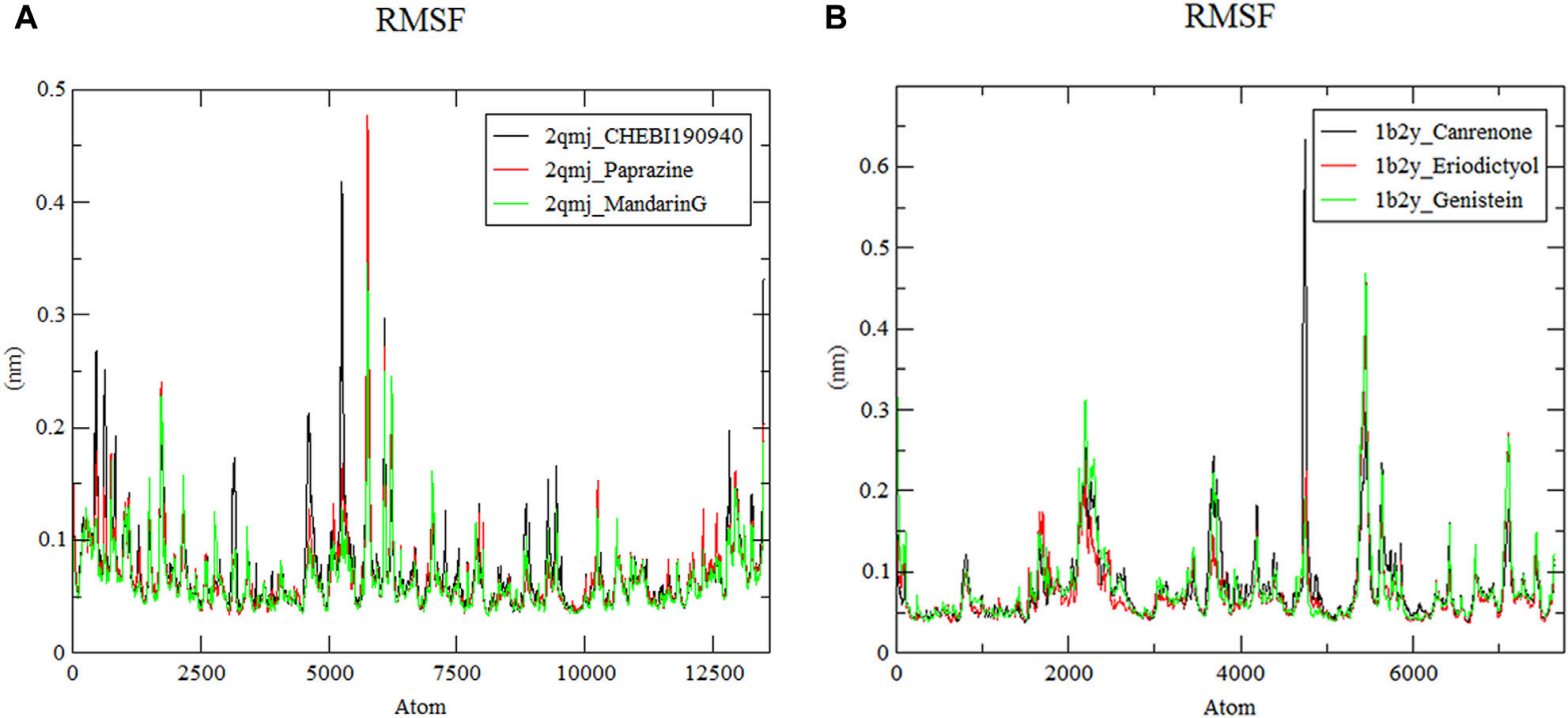
Root mean square fluctuation of ligand−protein systems. **(A)** 2qmj is *α*-glucosidase, Ligand is CHEBI:190940, paprazine and mandarinG **(B)** 1b2y is *α*-amylase, Ligand is canrenone, eriodictyol and genistein.

As displayed in Figure 7, RMSF represents the shift of each residue in α-glucosidase and α-amylase within four docking systems (Wang R. et al., 2023). The *α*-amylase−eriodictyol and *α*-glucosidase−mandarinG complex with an average RMSF value of 0.077 nm and 0.067 nm had more stable residues than *α*-amylase−canrenone, *α-*amylase−genistein (with RMSF values of 0.085 nm and 0.083 nm, respectively) and *α*-glucosidase−CHEBI190940, *α*-glucosidase−paprazine (with RMSF values of 0.077 nm and 0.071 nm, respectively). However, all docking systems displayed a similar fluctuation trend. Notably, irregular structures at amino acid residues between positions 4,500–6,500 led to abrupt fluctuations; nevertheless, these systems exhibited stability and cohesive connections within a simulated human environment despite some dynamic shifts.

#### 3.8.3 Radius of gyration analysis

**FIGURE 8:**
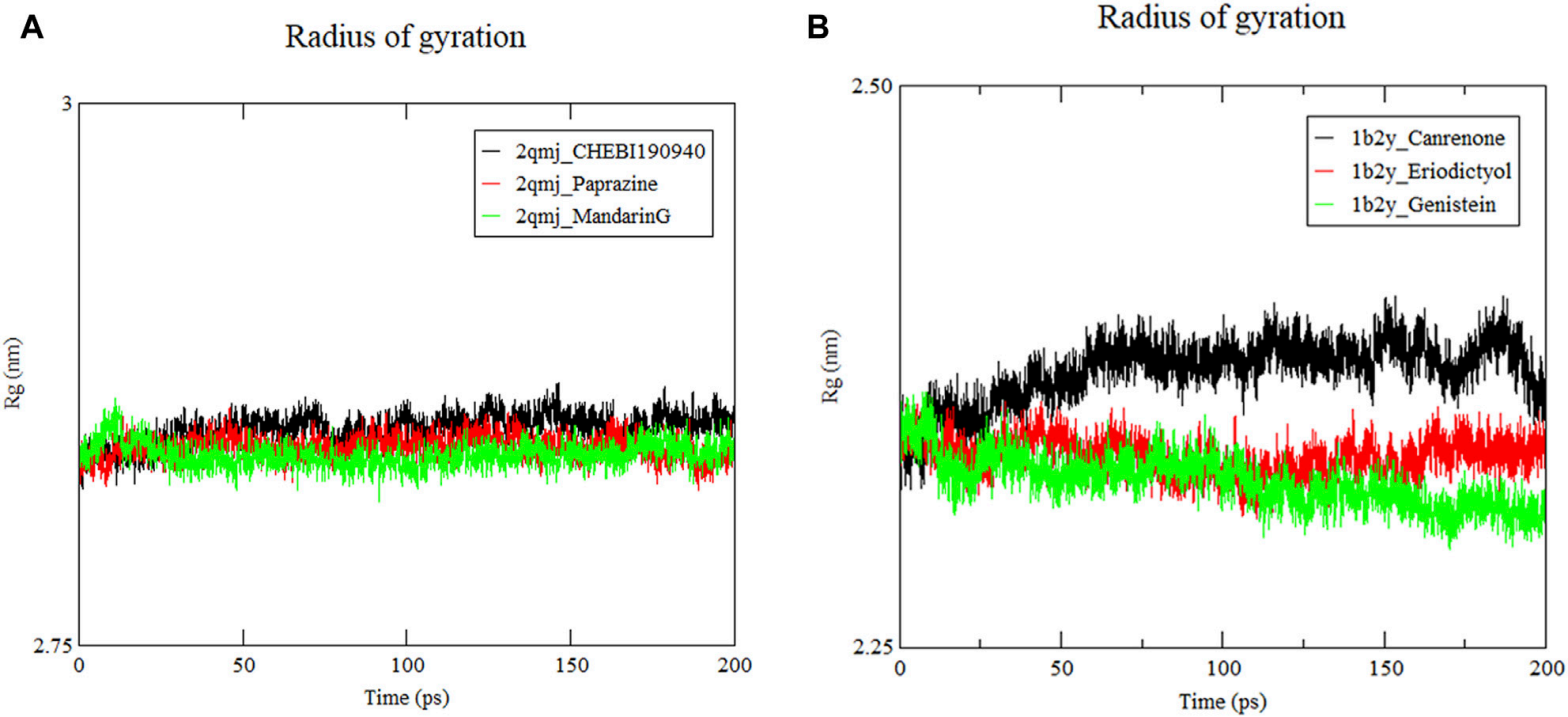
Radius of gyration (Rg) of ligand−protein systems. **(A)** 2qmj is *α*-glucosidase, Ligand is CHEBI:190940, paprazine and mandarinG **(B)** 1b2y is *α*-amylase, Ligand is canrenone, eriodictyol and genistein.

Figure 8 displays the Rg the complexes, where a smaller Rg value signifies a more compact structure (Chouhan et al., 2021). Stability across all docking systems was attained after 50 ns of simulation. The compactness of the structures ranked from most to least compact as follows: *α*-amylase−genistein, *α*-amylase−eriodictyol and *α*-amylase−canrenone. Their average Rg values were 2.32 nm, 2.34 nm, and 2.37 nm, respectively. respectively. These values are comparable to the previously reported Rg of 2.40 nm for α-glucosidase peptides, suggesting similar structural stability (Zheng et al., 2023). As shown in Figures 8A, B, the Rg value of *α*-glucosidase−mandarinG remained stable around 0.25 nm throughout the simulation. The Rg values of *α*-glucosidase−CHEBI190940 and *α*-glucosidase−paprazine fluctuated moderately between 2.85 nm and 2.84 nm, respectively. Among these peptides, the densest system was the *α*-amylase−eriodictyol and *α*-glucosidase−mandarinG complex with high conformational stability, aligning with the RMSD and RMSF results.

## 4 Discussion

DM is a prevalent chronic metabolic disease globally, often leading to severe complications. Presently, the primary pharmacological treatments for DM rely on chemical−based drugs, which exhibit limited long−term efficacy and may induce adverse reactions. As a folk medicine, the hypoglycemic activity of corn silk has been widely reported (Guo et al., 2009; Chang et al., 2016; Wang and Zhao, 2019; Hamzah et al., 2021; Wans et al., 2021). Nonetheless, as by−products of corn, there is a notable lack of systematic researches on straw peels and straw core, particularly regarding their anti−diabetic effects and impact on associated complications. Using a simple process to develop health-beneficial straw peels and straw core fractions would enhance the utilization of corn by-products, which are often discarded as agricultural waste. Preliminary findings indicate that CSE, SPE, and SCE can effectively inhibit activities of α-glucosidase and α-amylase, with SPE showing the most pronounced efficacy (Yang et al., 2021a). The management of DM and its associated complications often involves the regulation of postprandial hyperglycemia through the inhibition of *α*-amylase and *α*-glucosidase, which effectively modulating glucose absorption (Wang et al., 2010). Notably, inhibiting α-glucosidase can effectively control postprandial hyperglycemia (Shah et al., 2020). Natural products with distinct inhibitory activities against these enzymes have garnered increased interest in the management of DM, owing to their efficacy, safety, and minimal toxicity. Specifically, plant extracts rich in phenolics and flavonoids have been identified as potent *α*-amylase and *α*-glucosidase inhibitors (Tadera et al., 2006; Ademiluyi and Oboh, 2012). In this study, SPE was found to contain the highest levels of phenolics and flavonoids compared to CSE and SCE, with concentrations measured as rutin equivalent (182.3 ± 0.16) mg/g and gallic acid equivalent (215.1 ± 0.91) mg/g, respectively. The anti−hyperglycemic activities of SPE demonstrated a positive correlation with their TPC and TFC, aligning with findings from previous studies on Rheum turkestanicum (Dehghan et al., 2018). These results highlight the hypoglycemic potential of corn straw, underscoring the varying contributions of polar components from different sections of corn to DM management.

OS is closely linked to DM and its complications (Darenskaya et al., 2021). It originates from an imbalance between excessive free radical production and inadequate antioxidant defense. Among phytochemical antioxidants, phenolic compounds and flavonoids are distinguished for their ability to neutralize free radicals through hydrogen atom or electron donation (Aryal et al., 2019). Notably, the previous study reported that corn silk’s ethyl acetate extract had the highest antioxidant activity and total phenolic/flavonoid contents compared to petroleum ether and ethanol extracts (Wang and Zhao, 2019). In comparative assessments using DPPH, ABTS, •OH, and FRAP assays, SPE emerged as the most effective antioxidant, especially in free radical scavenging, likely due to its superior phenolic and flavonoid concentrations.

IR refers to the diminished efficacy of insulin in influencing its target tissues, commonly observed in individuals with metabolic disorders such as obesity (Kullmann et al., 2016). The elevation in insulin demand, prompted by IR, intensifies the development of T2DM and complicates the management of blood glucose levels among diabetic patients (Szoke and Gerich, 2005; Brown et al., 2010; Kampmann et al., 2011). When IR occurs, the expression of enzymes associated with hepatic glycolysis, such as GK and G−6−P, experiences significant modifications. These alterations precipitate a diminished capacity for glucose consumption and utilization by target cells, ultimately disrupting blood glucose homeostasis. Our findings demonstrate that within an IR model established by adding 10^–7^ mol/L insulin to HepG2, there was a discernible decrease in GK content, accompanied by an elevation in G−6−P levels. Subsequent treatment with SPE, CSE, and SCE in this IR−HepG2 model showed increased GK levels and reduced G−6−P levels, notably with SPE at 0.5 mg/mL significantly normalizing the expressions of GK and G−6−P to closely match those observed in the positive control group. These findings suggest that SPE significantly promotes glucose consumption in IR−HepG2 in a dose−dependent manner, while positively affecting glycolysis and glucose metabolism processes.

In the context of diabetes, sustained hyperglycemia inflicts damage on vascular endothelial cells, rendering them to pathological changes (Wang J. et al., 2023). This damage alters cytokine secretion, potentially leading to serious cerebrovascular and cardiovascular conditions, including cerebral hemorrhage, cerebral infarction, and coronary artery disease, mediated through alterations in vasodilation and the fibrinolytic system (Zhang et al., 1998; Shen, 2010). NO and ET−1 serve as critical regulators of vascular dilation, with ET−1 acting as a potent vasoconstrictor (Spieker et al., 2001; Hynynen and Khalil, 2006), whereas NO facilitates vascular relaxation (Giles, 2006). Under normal physiological conditions, the synthesis of these molecules is counterbalanced, maintaining a dynamic equilibrium essential for vascular systolic and diastolic functions (Goto et al., 1996; Zhao et al., 2010). Additionally, t−PA and PAI−1 regulate the blood coagulation process, with t−PA activating plasminogen and PAI−1 inhibiting the effect of t−PA by forming a complex with it (Wagner et al., 1989; Pandolfi et al., 1996). In the present study, we demonstrated that SPE significantly upregulates NO and t−PA levels while downregulating ET−1 and PAI−1 expressions, suggesting that SPE ameliorates hyperglycemia−induced endothelial damage by promoting endothelium−dependent vasodilation and fibrinolytic function.

This study employed UPLC−QE−Orbitrap−MS technology for a swift and comprehensive analysis of the chemical constituents within SPE, CSE, and SCE, leading to the identification of 61 compounds, including vanillic acid, gallic acid, gentisic acid, naringenin, genistein, canrenone, eriodictyol, and CHEBI190940. Prior research has demonstrated the potent inhibitory effects of phenolic compounds, such as vanillic acid, gallic acid, gentisic acid, naringenin, and genistein, on α-amylase and *α*-glucosidase (Ahmad et al., 2021; Osman et al., 2021; Aleixandre et al., 2022; El Karkouri et al., 2022). Several polyphenols, such as protocatechuic acid and naringenin, have been shown to enhance glucose consumption in IR−HepG2. Protocatechuic acid facilitates glucose consumption by activating AMPK phosphorylation and upregulating glucose transporter 4 (GLUT4) expression (Yang X. Y. et al., 2021). Naringenin enhances AMPK phosphorylation at Thr172, stimulating glucose transporter 2 (GLUT2) translocation and thereby improving glucose consumption in IR−HepG2 (Dayarathne et al., 2021). Moreover, eriodictyol primarily boosts insulin−sensitive glucose consumption through activation of the PI3K/Akt signaling pathway (Zhang et al., 2012). Canrenone is noted for its regulatory effects on fasting plasma glucose levels and blood pressure (Derosa et al., 2018). This study marks the first identification of canrenone within SPE, specifically from the *Zea* L. species, expanding the knowledge on the bioactive components of SPE and their potential therapeutic implications.

Following this, we conducted *in silico* virtual docking studies of the identified compounds with the crystal structures of *α*-glucosidase and *α*-amylase using AutoDock, employing acarbose as a positive control due to its recognized binding to these enzymes. The outcomes suggest these candidate compounds can potentially interact with the active sites of *α*-amylase and *α*-glucosidase, thus inhibiting their enzymatic functions. Notably, docking simulations indicated canrenone possessed the highest binding affinity toward *α*-amylase, surpassing acarbose, by effectively occupying the enzyme’s active site. This can be attributed to canrenone’s strong hydrogen bond interactions with the HIS−201 and GLN−63 residues, yielding a higher binding energy. Similarly, CHEBI190940 exhibited a stronger binding interaction (−7.8 kcal/mol) with *α*-glucosidase compared to acarbose (7.3 kcal/mol), particularly due to significant interactions with the catalytic residues ASP−327 and ASP−205, leading to a marked reduction in *α*-glucosidase activity.In essence, the *in vitro* hypoglycemic activity observed in CSE, SPE, and SCE could be attributed to the inhibition of α-glucosidase and *α*-amylase, alongside the promotion of glucose consumption in IR−HepG2.

Molecular dynamics simulation is a potent tool for elucidating the stability and dynamics of protein−ligand complexes, crucial for drug design (Leeson and Young, 2015). They enable the identification of structural cavities essential for developing new compounds with superior target affinity. Molecular dynamics simulations facilitate the acquisition of detailed structural information and insights into how protein stability influences ligand binding. This leads to improved sampling of binding poses and more precise estimates of binding affinity, enhancing structural precision (Yunta, 2017). Higher values of RMSD, RMSF and Rg indicate increased system flexibility (Zhang et al., 2017). Our molecular dynamics simulations on docking complexes aim to investigate structural alterations upon ligand interaction. After 50 ns, the amylase and glucosidase complexes reached stability, with RMSD values below 0.3, signifying stable complexes. Concurrently, Rg and RMSF values further denote the complexes’ stability. Such computational analyses provide vital insights for identifying viable medication candidates for T2DM management. However, the path to new drug development requires consideration of pharmacokinetic attributes, therapeutic efficacy, and safety profiles (Doss et al., 2013; Peele et al., 2020). Specifically, molecular docking and absorption, distribution, metabolism, excretion, and toxicity (ADMET) analysis of phytochemicals (P. betle compounds) have exhibited a wide range of physicochemical, pharmacokinetic, and drug−likeness properties, positioning in−silico screening as a viable method for discovering new therapeutics (Wang and Urban, 2004) from natural sources (Peele et al., 2020).

## 5 Conclusion

In summary, our study demonstrates that SPE, SCE, and CSE are abundant in natural hypoglycemic agents and antioxidants, particularly flavonoids and phenolics, with their concentrations ranking in the order of SPE > SCE > CSE for both total flavonoids and phenolics. Initial assays on *α*-glucosidase and *α*-amylase inhibition validated this efficacy hierarchy. Moreover, glucose consumption assays revealed a substantial increase in glucose absorption by IR−HepG2 treated with SPE. Analytical outcomes from UPLC−QE−Orbitrap−MS and molecular docking, further substantiated by molecular dynamics simulations, verified interactions between candidate compounds (including canrenone, CHEBI190940, erodictyol, paprazine, genistein, and mandarin G) and the target *α*-glucosidase and *α*-amylase. These findings are consistent with the antioxidation capacities assessed by DPPH, FRAP, ABTS, and •OH scavenging assays, which also follow the sequence of SPE > SCE > CSE. The enhanced antioxidant capacity of SPE is attributed to its elevated levels of flavonoids and phenolics, while its hypoglycemic effect likely stems from the inhibition of *α*-glucosidase and *α*-amylase activities, alongside facilitating glucose consumption in IR−HepG2 and providing protection against high glucose−induced damage in HUVECs. This research suggests that SPE and/or SCE could be promising natural sources for hypoglycemic and antioxidant interventions in nutraceutical and functional food development, presenting an innovative strategy for the sustainable utilization of corn by−products.

## Data Availability

The original contributions presented in the study are included in the article/Supplementary Material, further inquiries can be directed to the corresponding authors.
