# Supplementary material for: From waste to wonder: exploring the hypoglycemic and anti-oxidant properties of corn processing by−products
Source: Front Chem. 2024 Jul 18;12:1433501. doi: 10.3389/fchem.2024.1433501 (PMC11299435; doi:10.3389/fchem.2024.1433501)
Supplement: Supplementary file 1 [file DataSheet1.docx]

Supplementary Material

# 1 Anti−Insulin resistance activity

HepG2 were cultured in a 6-well plate at a density of 5×104/mL and incubated in MEM supplemented with 10% fetal bovine serum, 1% (v/v) penicillin-streptomycin solution. The incubation was carried out at 37°C in a humidified incubator with an atmosphere containing 5% CO2. In order to establish an in vitro model of insulin resistance (IR), HepG2 were treated with insulin. Initially, insulin was diluted to a concentration of 10−7 mol/L using the whole-cell culture medium, which was then added to the culture plate and further incubated for 24 h. Fresh insulin solutions were prepared immediately before each experiment and incubated at 37°C in a CO2 enriched, humidified atmosphere throughout the incubation period. Following this, SPE, SCE, and CSE extracts at concentrations of (0.125, 0.25, 0.5 mg/mL) were added to the cells and allowed to react for another 24 h at a temperature of 37°C. Metformin served as the positive control. Blank medium served as control. Three duplicate wells in each group were set up. The cell−free supernatants were collected to determine the glucose content levels using the glucose assay kit (Nanjing Jiancheng Bioengineering Institute, Nanjing, China), Cells underwent triple washes with pre−cooled phosphate buﬀer (PBS) and subsequent digestion with 0.25% trypsin until they exhibited shrinkage and rounding. The digestion process was halted by supplementing with an equal volume of serum, followed by centrifugation at 1000 rpm for 5 min. Post centrifugation, cells were washed thrice using PBS, rapidly frozen in liquid nitrogen, and then stored at −80°C overnight. Subsequent thawing of both cells and culture medium occurred in a 4°C refrigerator, with the samples undergoing five freeze−thaw cycles between −80°C and 4°C. For cellular content release, cells were vortexed in PBS, followed by ultrasonic disruption in an ice bath (5 sec on, 9 sec off, for a duration of 20 min). Finally, the samples were centrifuged at 13,000 rpm (14000 rpm in a FA-45-30-11 rotor, Centrifuge 5430 R, Eppendorf, Germany) at 4°C for 10 min, the supernatant was carefully transferred into a centrifuge tube, ready for determine the Glucokinase (GK), Glucose−6−phosphatase (G−6−P) levels using the ELISA assay kits (Shanghai Huiying Biological Technology Co., Ltd., Shanghai, China).

# 2 HUVECs damage induced by high glucose

HUVECs (5×104/mL) were seeded in 6−wells culture plate and incubated in DMEM supplemented with 10% fetal bovine serum, 1% (v/v) penicillin–streptomycin solution at 37°C in an atmosphere of 5% CO2 in a humidified incubator. To establish a model of endothelial dysfunction in vitro, High glucose was used on HUVECs. First, High glucose was diluted to 40 mmol/L with whole cell culture medium, then added to a culture plate and incubated for 24 h. All glucose solutions were made fresh before use. and incubated at 37°C for 24 h in an atmosphere of CO2 in a humidified incubator. After incubation,100 µL of (0.125, 0.25, 0.5 mg/mL) of SPE, SCE and CSE extract was added and reacted at 37°C for 24 h. Rosiglitazone served as the positive control. Blank medium served as control. Three duplicate wells in each group were set up. Cells underwent triple washes with precooled PBS and subsequent digestion with 0.25% trypsin until they exhibited shrinkage and rounding. The digestion process was halted by supplementing with an equal volume of serum, followed by centrifugation at 1000 rpm for 5 min. Post centrifugation, cells were washed thrice using PBS, rapidly frozen in liquid nitrogen, and then stored at −80°C overnight. Subsequent thawing of both cells and culture medium occurred in a 4°C refrigerator, with the samples undergoing five freeze thaw cycles between −80°C and 4°C. For cellular content release, cells were vortexed in PBS, followed by ultrasonic disruption in an ice bath (5 sec on, 9 sec off, for a duration of 20 min). Finally, after centrifugation at 4°C and 13000 rpm for 10 min, the supernatant was carefully transferred into a centrifuge tube, ready for determine the tissue−type plasminogen activator (t−PA), endothelin−1 (ET−1), nitric oxide (NO) and plasminogen activator inhibitor 1 (PAI−1) levels were detected with ELISA assay kits (Shanghai Huiying Biological Technology Co., Ltd., Shanghai, China).

# 3 Supplementary Tables

Supplementary Table 1. Cell viability assays results of SPE, SCE and CSE.

|  | **Control** | | | **SPE** | | | **SCE** | | | **CSE** | | |
| --- | --- | --- | --- | --- | --- | --- | --- | --- | --- | --- | --- | --- |
| Control | 96.57 | 97.15 | 100.29 |  |  |  |  |  |  |  |  |  |
| 0.125 mg/mL |  |  |  | 93.20 | 93.42 | 99.39 | 98.34 | 87.52 | 91.14 | 95.45 | 93.70 | 97.85 |
| 0.25 mg/mL |  |  |  | 88.82 | 89.84 | 95.35 | 89.47 | 98.64 | 93.89 | 92.16 | 87.93 | 94.91 |
| 0.5 mg/mL |  |  |  | 100.77 | 101.94 | 108.28 | 104.41 | 101.03 | 94.57 | 105.31 | 102.11 | 94.58 |

Supplementary Table 2. Glucose consumption of SPE, SCE and CSE.

|  | **Control** | | | **Model** | | | **Metformin** | | | **SPE** | | | **SCE** | | | **CSE** | | |
| --- | --- | --- | --- | --- | --- | --- | --- | --- | --- | --- | --- | --- | --- | --- | --- | --- | --- | --- |
| Control | 10.00 | 11.00 | 10.47 |  |  |  |  |  |  |  |  |  |  |  |  |  |  |  |
| Model |  |  |  | 7.30 | 7.90 | 8.40 |  |  |  |  |  |  |  |  |  |  |  |  |
| Metformin |  |  |  |  |  |  | 13.90 | 14.10 | 14.36 |  |  |  |  |  |  |  |  |  |
| 0.125 mg/mL |  |  |  |  |  |  |  |  |  | 7.95 | 8.89 | 8.00 | 7.25 | 8.95 | 8.35 | 7.50 | 8.50 | 8.27 |
| 0.25 mg/mL |  |  |  |  |  |  |  |  |  | 9.50 | 10.20 | 9.10 | 8.80 | 9.40 | 9.30 | 8.65 | 9.70 | 8.98 |
| 0.5 mg/mL |  |  |  |  |  |  |  |  |  | 10.70 | 11.20 | 10.50 | 9.50 | 10.60 | 9.35 | 9.00 | 10.20 | 9.63 |

Supplementary Table 3. The outcomes of treatment with SPE, SCE, and CSE on the GK activity in IR−HepG2.

|  | **Control** | | | **Model** | | | **Metformin** | | | **SPE** | | | **SCE** | | | **CSE** | | |
| --- | --- | --- | --- | --- | --- | --- | --- | --- | --- | --- | --- | --- | --- | --- | --- | --- | --- | --- |
| Control | 228.20 | 234.80 | 222.45 |  |  |  |  |  |  |  |  |  |  |  |  |  |  |  |
| Model |  |  |  | 103.35 | 99.10 | 113.60 |  |  |  |  |  |  |  |  |  |  |  |  |
| Metformin |  |  |  |  |  |  | 215.60 | 217.70 | 223.42 |  |  |  |  |  |  |  |  |  |
| 0.125 mg/mL |  |  |  |  |  |  |  |  |  | 125.20 | 155.90 | 140.55 | 121.85 | 132.85 | 145.85 | 122.30 | 140.00 | 131.10 |
| 0.25 mg/mL |  |  |  |  |  |  |  |  |  | 158.34 | 180.28 | 169.10 | 145.27 | 175.27 | 160.27 | 149.00 | 169.00 | 159.78 |
| 0.5 mg/mL |  |  |  |  |  |  |  |  |  | 185.70 | 205.92 | 186.50 | 174.80 | 195.00 | 188.00 | 173.5 | 185.00 | 178.65 |

Supplementary Table 4. The outcomes of treatment with SPE, SCE, and CSE on the G-6-P activity in IR−HepG2.

|  | **Control** | | | **Model** | | | **Metformin** | | | **SPE** | | | **SCE** | | | **CSE** | | |
| --- | --- | --- | --- | --- | --- | --- | --- | --- | --- | --- | --- | --- | --- | --- | --- | --- | --- | --- |
| Control | 118.00 | 122.50 | 120.75 |  |  |  |  |  |  |  |  |  |  |  |  |  |  |  |
| Model |  |  |  | 210.50 | 224.75 | 218.00 |  |  |  |  |  |  |  |  |  |  |  |  |
| Metformin |  |  |  |  |  |  | 127.50 | 130.25 | 133.00 |  |  |  |  |  |  |  |  |  |
| 0.125 mg/mL |  |  |  |  |  |  |  |  |  | 158.00 | 181.09 | 171.18 | 171.20 | 179.80 | 187.70 | 180.24 | 191.22 | 187.20 |
| 0.25 mg/mL |  |  |  |  |  |  |  |  |  | 145.50 | 167.00 | 158.30 | 150.50 | 171.05 | 161.92 | 167.11 | 186.13 | 172.10 |
| 0.5 mg/mL |  |  |  |  |  |  |  |  |  | 133.20 | 149.30 | 141.10 | 142.47 | 153.01 | 139.03 | 155.25 | 157.20 | 150.27 |

Supplementary Table 5. The outcomes of treatment with SPE, SCE, and CSE on the NO activity in HUVECs.

|  | **Control** | | | **Model** | | | **Rosiglitazone** | | | **SPE** | | | **SCE** | | | **CSE** | | |
| --- | --- | --- | --- | --- | --- | --- | --- | --- | --- | --- | --- | --- | --- | --- | --- | --- | --- | --- |
| Control | 122.40 | 129.05 | 125.80 |  |  |  |  |  |  |  |  |  |  |  |  |  |  |  |
| Model |  |  |  | 69.12 | 70.89 | 72.45 |  |  |  |  |  |  |  |  |  |  |  |  |
| Rosiglitazone |  |  |  |  |  |  | 94.45 | 105.92 | 101.13 |  |  |  |  |  |  |  |  |  |
| 0.125 mg/mL |  |  |  |  |  |  |  |  |  | 80.16 | 86.79 | 82.91 | 79.47 | 86.12 | 80.35 | 79.21 | 80.95 | 82.78 |
| 0.25 mg/mL |  |  |  |  |  |  |  |  |  | 86.32 | 96.45 | 89.97 | 82.55 | 77.75 | 88.90 | 76.21 | 87.95 | 83.78 |
| 0.5 mg/mL |  |  |  |  |  |  |  |  |  | 99.02 | 95.87 | 97.93 | 88.23 | 92.15 | 89.97 | 85.94 | 92.56 | 88.72 |

Supplementary Table 6. The outcomes of treatment with SPE, SCE, and CSE on the ET-1 activity in HUVECs.

|  | **Control** | | | **Model** | | | **Rosiglitazone** | | | **SPE** | | | **SCE** | | | **CSE** | | |
| --- | --- | --- | --- | --- | --- | --- | --- | --- | --- | --- | --- | --- | --- | --- | --- | --- | --- | --- |
| Control | 99.30 | 96.21 | 94.99 |  |  |  |  |  |  |  |  |  |  |  |  |  |  |  |
| Model |  |  |  | 131.76 | 139.62 | 138.92 |  |  |  |  |  |  |  |  |  |  |  |  |
| Rosiglitazone |  |  |  |  |  |  | 103.51 | 96.47 | 99.22 |  |  |  |  |  |  |  |  |  |
| 0.125 mg/mL |  |  |  |  |  |  |  |  |  | 92.38 | 92.32 | 98.09 | 99.77 | 96.82 | 103.31 | 103.41 | 100.75 | 98.85 |
| 0.25 mg/mL |  |  |  |  |  |  |  |  |  | 96.62 | 91.53 | 92.55 | 102.71 | 98.32 | 95.17 | 100.69 | 94.19 | 97.12 |
| 0.5 mg/mL |  |  |  |  |  |  |  |  |  | 90.67 | 90.52 | 94.51 | 99.59 | 95.98 | 96.03 | 96.93 | 92.98 | 98.29 |

Supplementary Table 7. The outcomes of treatment with SPE, SCE, and CSE on the PAI−1 activity in HUVECs.

|  | **Control** | | | **Model** | | | **Rosiglitazone** | | | **SPE** | | | **SCE** | | | **CSE** | | |
| --- | --- | --- | --- | --- | --- | --- | --- | --- | --- | --- | --- | --- | --- | --- | --- | --- | --- | --- |
| Control | 94.48 | 95.97 | 98.05 |  |  |  |  |  |  |  |  |  |  |  |  |  |  |  |
| Model |  |  |  | 135.83 | 132.75 | 138.72 |  |  |  |  |  |  |  |  |  |  |  |  |
| Rosiglitazone |  |  |  |  |  |  | 110.34 | 104.84 | 101.02 |  |  |  |  |  |  |  |  |  |
| 0.125 mg/mL |  |  |  |  |  |  |  |  |  | 99.97 | 107.74 | 100.08 | 107.50 | 106.67 | 101.73 | 107.93 | 103.47 | 106.60 |
| 0.25 mg/mL |  |  |  |  |  |  |  |  |  | 93.27 | 91.97 | 88.47 | 105.75 | 104.11 | 102.15 | 102.10 | 98.89 | 96.02 |
| 0.5 mg/mL |  |  |  |  |  |  |  |  |  | 84.26 | 86.03 | 91.46 | 91.22 | 93.38 | 90.90 | 96.11 | 91.76 | 88.13 |

Supplementary Table 8. The outcomes of treatment with SPE, SCE, and CSE on the t-PA activity in HUVECs.

|  | **Control** | | | **Model** | | | **Rosiglitazone** | | | **SPE** | | | **SCE** | | | **CSE** | | |
| --- | --- | --- | --- | --- | --- | --- | --- | --- | --- | --- | --- | --- | --- | --- | --- | --- | --- | --- |
| Control | 46.75 | 48.75 | 46.50 |  |  |  |  |  |  |  |  |  |  |  |  |  |  |  |
| Model |  |  |  | 30.90 | 29.90 | 28.90 |  |  |  |  |  |  |  |  |  |  |  |  |
| Rosiglitazone |  |  |  |  |  |  | 42.52 | 41.53 | 41.95 |  |  |  |  |  |  |  |  |  |
| 0.125 mg/mL |  |  |  |  |  |  |  |  |  | 37.83 | 38.57 | 37.69 | 38.78 | 38.35 | 36.87 | 37.49 | 35.77 | 35.84 |
| 0.25 mg/mL |  |  |  |  |  |  |  |  |  | 42.96 | 42.76 | 41.48 | 39.94 | 38.79 | 39.77 | 37.87 | 38.93 | 38.01 |
| 0.5 mg/mL |  |  |  |  |  |  |  |  |  | 46.15 | 46.93 | 46.42 | 45.61 | 44.78 | 45.31 | 40.13 | 40.89 | 39.08 |

Supplementary Table 9. UPLC−QE−Orbitrap−MS analytical results of composition for the SPE, SCE and CSE

| **NO.** | **Identification** | **Rt**  **(min)** | **Measured**  **(m/z)** | **Calculated**  **(m/z)** | **Error**  **(ppm)** | **Molecular**  **formula** | **MS^2^**  **fragments** | **Ion form** | **Source** | **References** |
| --- | --- | --- | --- | --- | --- | --- | --- | --- | --- | --- |
| 1 | Vanillic acid | 3.82 | 168.0423 | 168.0423 | 0.00 | C_8_H_8_O_4_ | 167.0346,  152.0111,  150.9689,  108.0206 | [M−H] ^−^ | SPE,  SCE,  CSE | (Simayi et al., 2022) |
| 2 | Gallic acid | 15.57 | 170.1206 | 170.1200 | 3.53 | C_7_H_6_O_5_ | 125.0963,  107.0241,  97.0284,  79.3119 | [M−H] ^−^ | SPE,  SCE,  CSE | (Singh et al., 2016) |
| 3 | Protocatechuic acid | 26.90 | 154.0264 | 154.0266 | −1.30 | C_7_H_6_O_4_ | 153.0189,  137.0187,  107.4791 | [M−H] ^−^ | SPE,  SCE,  CSE | (Balkrishna et al., 2022) |
| 4 | Malic acid | 0.53 | 134.0214 | 134.0215 | −0.75 | C_4_H_6_O_5_ | 115.0029,  89.0234,  71.0127 | [M−H] ^−^ | SPE,  SCE,  CSE | (Câmara Neto et al., 2022) |
| 5 | 4−Hydroxybenzaldehyde | 0.36 | 122.0365 | 122.0368 | −2.46 | C_7_H_6_O_2_ | 121.0287  92.9589 | [M−H] ^−^ | SPE,  SCE,  CSE | (Jia et al., 2014) |
| 6 | *p*−coumaric acid | 18.41 | 164.0473 | 164.0473 | 0.00 | C_9_H_8_O_3_ | 163.0396,  149.8434,  120.0527,  119.0494 | [M−H] ^−^ | SPE,  SCE,  CSE | (Yin et al., 2019) |
| 7 | Pimelic acid | 4.28 | 160.0738 | 160.0736 | 1.25 | C_7_H_12_O_4_ | 159.0658,  130.9828,  115.0757,  114.0916,  102.9881,  97.0650 | [M−H] ^−^ | SPE,  SCE,  CSE | (Yin et al., 2019) |
| 8 | Hydroxybenzoic acid | 26.31 | 154.1204 | 154.1210 | −3.89 | C_7_H_6_O_4_ | 153.8682,  109.0287,  100.8701 | [M−H] ^−^ | SPE,  SCE,  CSE | (Peixoto et al., 2021) |
| 9 | Suberic acid | 7.66 | 174.0894 | 174.0892 | 1.15 | C_8_H_14_O_4_ | 173.0817,  111.0807 | [M−H] ^−^ | SPE,  SCE,  CSE | (Kasiotis et al., 2023) |
| 10 | *p*−Anisic acid | 5.70 | 152.0463 | 152.0473 | −6.58 | C_8_H_8_O_3_ | 151.0397,  106.0403 | [M−H] ^−^ | SPE,  CSE | (Bashar et al., 2022) |
| 11 | 3,4−Dihydroxyphenylacetic  acid | 3.27 | 168.1474 | 168.1480 | −3.57 | C_8_H_8_O_4_ | 123.0444,  122.0321,  108.0209 | [M−H] ^−^ | SPE,  SCE,  CSE | (Yan et al., 2020) |
| 12 | 3−hydroxyphenylacetic  acid | 1.48 | 152.0463 | 152.0473 | −6.58 | C_8_H_8_O_3_ | 121.0290,  107.0494,  93.0336 | [M−H] ^−^ | SPE,  SCE,  CSE | (Yan et al., 2020) |
| 13 | 4−hydroxybenzoic acid | 8.08 | 138.0307 | 138.0317 | −7.24 | C_7_H_6_O_3_ | 93.0335 | [M−H] ^−^ | SCE,  CSE | (Yan et al., 2020) |
| 14 | Fenoprofen | 12.78 | 242.0922 | 242.0943 | 8.67 | C_15_H_14_O_3_ | 197.1182 | [M−H] ^−^ | SPE,  SCE,  CSE | (Bharwad et al., 2020) |
| 15 | Sebacic Acid | 12.66 | 202.1219 | 202.1205 | −6.93 | C_10_H_18_O_4_ | 183.1022,  139.1122 | [M−H] ^−^ | SPE,  SCE,  CSE | (Bi et al., 2017) |
| 16 | (+/−)9−HpODE | 19.05 | 312.2314 | 312.2301 | 4.16 | C_18_H_32_O_4_ | 201.1133,  197.5171,  171.1022 | [M−H] ^−^ | SPE | (Popa et al., 2021) |
| 17 | 2,3−Dihydroxybenzoic acid | 7.01 | 154.1204 | 154.1210 | −3.89 | C_7_H_6_O_4_ | 108.0527,  80.0257 | [M−H] ^−^ | SPE  CSE | (Wroblewska et al., 2019) |
| 18 | 4−Nitrophenol | 7.90 | 139.0269 | 139.0269 | 0.00 | C_6_H_5_NO_3_ | 108.0209,  92.9190 | [M−H] ^−^ | SPE,  SCE,  CSE | (Pu et al., 2018) |
| 19 | Palmitic acid | 13.63 | 254.2386 | 254.2402 | −6.24 | C_16_H_30_O_2_ | 235.1342,  253.1445 | [M−H] ^−^ | CSE | (Zhao et al., 2021) |
| 20 | Azelaic acid | 10.06 | 188.1042 | 188.1049 | −3.72 | C_9_H_16_O_4_ | 169.0868  143.1071,  125.0965,  123.0809,  97.0649,  57.0335 | [M−H] ^−^ | SPE,  SCE,  CSE | (Lu et al., 2021) |
| 21 | 2−Hydroxyphenylacetic acid | 1.67 | 152.0463 | 152.0473 | −6.58 | C_8_H_8_O_3_ | 151.0397,  107.0493 | [M−H] ^−^ | SPE,  SCE,  CSE | (Kajita et al., 1993) |
| 22 | Phenylglyoxylic acid | 32.04 | 150.0307 | 150.0317 | −6.67 | C_8_H_6_O_3_ | 121.0289,  105.0242,  77.3148 | [M−H] ^−^ | SPE,  SCE | (Marchese et al., 2004) |
| 23 | Gentisic acid | 29.29 | 154.0264 | 154.0266 | −1.30 | C_7_H_6_O_4_ | 153.8681,  109.0288 | [M−H] ^−^ | SPE,  SCE,  CSE | (Bai et al., 2019) |
| 24 | Naringenin | 14.24 | 272.0674 | 272.0685 | −4.04 | C_15_H_12_O_5_ | 177.0192,  151.0031,  119.0494,  107.0130,  93.0336 | [M−H] ^−^ | SPE,  SCE,  CSE | (Zeng et al., 2018) |
| 25 | Sakuranetin | 10.75 | 286.0859 | 286.0841 | 6.29 | C_16_H_14_O_5_ | 165.0191,  119.0494, | [M−H] ^−^ | SPE,  SCE, | (Zhou et al., 2023, Shen et al., 2019) |
| 26 | Eriodictyol | 12.50 | 288.0612 | 288.0634 | −7.64 | C_15_H_12_O_6_ | 151.0031  135.0446,  125.4798 | [M−H] ^−^ | SCE, | (Alvarez-Fernandez et al., 2015) |
| 27 | Gluconic acid | 11.50 | 196.0576 | 196.0583 | 3.57 | C_6_H_12_O_7_ | 177.2447,  129.7836 | [M−H] ^−^ | SPE | (Cadiz-Gurrea et al., 2013) |
| 28 | 4−Hydroxycinnamic acid | 7.57 | 164.0477 | 164.0473 | −2.44 | C_9_H_8_O_3_ | 119.0495,  93.0342 | [M−H] ^−^ | SPE,  SCE,  CSE | (Szewczyk et al., 2020) |
| 29 | Sinapaldehyde | 9.30 | 208.0739 | 208.0736 | −1.44 | C_11_H_12_O_4_ | 192.0427,  177.0191 | [M−H] ^−^ | SPE,  SCE | (Li et al., 2018) |
| 30 | (+/−)12(13) −DiHOME | 20.38 | 314.2433 | 314.2457 | −7.64 | C_18_H_34_O_4_ | 313.2390,  183.1388 | [M−H] ^−^ | SPE,  SCE,  CSE | (Kodani et al., 2022) |
| 31 | (+/−)9(10) −EpOME | 21.32 | 296.2339 | 296.2351 | −4.05 | C_18_H_32_O_3_ | 295.1760,  171.1027 | [M−H] ^−^ | SPE,  SCE,  CSE | (Kodani et al., 2022) |
| 32 | 3,5−Dihydroxybenzoic acid | 1.25 | 154.0264 | 154.0266 | −1.30 | C_7_H_6_O_4_ | 153.0188,  109.0286 | [M−H] ^−^ | SPE,  SCE,  CSE | (Santos et al., 2016) |
| 33 | 4−Hydroxyphenylacetic acid | 1.67 | 152.0463 | 152.0473 | −6.58 | C_8_H_8_O_3_ | 151.0397 ,107.0493 | [M−H] ^−^ | SPE,  SCE,  CSE | (Viacava et al., 2018) |
| 34 | Feruloyltyramine | 11.86 | 313.1307 | 313.1314 | −2.24 | C_18_H_19_NO_4_ | 178.0507,  148.0525,  135.0444 | [M−H] ^−^ | SPE,  SCE,  CSE | (Li et al., 2022) |
| 35 | Genistein | 15.75 | 270.0521 | 270.0528 | −2.59 | C_15_H_10_O_5_ | 159.5172,  134.0864 | [M−H] ^−^ | CSE | (Lin et al., 2020) |
| 36 | Salicylic acid | 2.35 | 138.0309 | 138.0317 | −5.8 | C_7_H_6_O_3_ | 93.0336,  75.5170 | [M−H] ^−^ | SPE,  SCE,  CSE | (Szewczyk et al., 2020) |
| 37 | Linoleic acid | 7.84 | 280.2418 | 280.2402 | 5.71 | C_18_H_32_O_2_ | 134.0371,  68.9658 | [M−H] ^−^ | SPE,  CSE | (Lee et al., 2022) |
| 38 | 2−Hydroxypalmitic acid | 14.24 | 272.2341 | 272.2351 | −3.67 | C_16_H_32_O_3_ | 253.7052,  225.3557, | [M−H] ^−^ | SPE,  CSE | (Glaser et al., 2020) |
| 39 | 9−hydroxy−10E,12Z−octadecadienoic acid | 21.02 | 296.2335 | 296.2351 | −5.40 | C_18_H_32_O_3_ | 295.2287  277.2183,  195.1391 | [M−H] ^−^ | SCE,  CSE | (Konczol et al., 2014) |
| 40 | Esculetin | 4.90 | 178.0269 | 178.0266 | 1.69 | C_9_H_6_O_4_ | 133.8923,  116.9649,  105.0338 | [M−H] ^−^ | SPE,  CSE | (Yang et al., 2017) |
| 41 | 3−(3,4−Dihydroxyphenyl) propionic acid | 22.87 | 182.0583 | 182.0579 | 2.20 | C_9_H_10_O_4_ | 137.3719,  109.7124 | [M−H] ^−^ | SPE,  SCE,  CSE | (Loo et al., 2022) |
| 42 | Mandarin G | 14.64 | 328.3403 | 328.342 | −5.18 | C_16_H_12_N_2_O_4_S | 327.2185,  172.5016 | [M−H] ^−^ | CSE | (Alam et al., 2023) |
| 43 | Canrenone | 21.84 | 340.2031 | 340.2038 | −3.23 | C_22_H_28_O_3_ | 339.2011,  183.0121 | [M−H] ^−^ | SPE | NA |
| 44 | 1−[2−(1,3−benzodioxol−5−yl) −3−methyl−1−benzofuran−5−yl] propane−1,2−diol (CHEBI:190940) | 21.22 | 326.3497 | 326.3480 | −5.21 | C_19_H_18_O_5_ | 278.2064,  183.0119 | [M−H] ^−^ | SPE | NA |
| 45 | Paprazine | 11.51 | 283.1225 | 283.1208 | −6.00 | C_17_H_17_NO_3_ | 162.0557,  119.0495 | [M−H] ^−^ | SPE,  SCE | NA |
| 46 | Isovanillic acid | 2.20 | 168.1474 | 168.148 | −3.57 | C_8_H_8_O_4_ | 152.0112,  123.0443,  108.0447 | [M−H] ^−^ | SPE,  SCE,  CSE | NA |
| 47 | Dihydroalbocycline | 19.06 | 310.2163 | 310.2144 | 6.12 | C_18_H_30_O_4_ | 185.1183,  163.1153,  99.0807 | [M−H] ^−^ | SCE | NA |
| 48 | Psoromic acid | 13.15 | 358.067 | 358.0689 | −5.31 | C_18_H_14_O_8_ | 348.3262,  339.0507,  332.1390,  332.1390 | [M−H] ^−^ | CSE | NA |
| 49 | *N*−Acetyl−*L*−tryptophan | 8.70 | 246.1019 | 246.1004 | −6.10 | C_13_H_14_N_2_O_3_ | 203.0828,  116.0346,  74.0238 | [M−H] ^−^ | SPE | NA |
| 50 | 3,9−Dihydroeucomin | 15.26 | 300.0980 | 300.0998 | −6.00 | C_17_H_16_O_5_ | 299.0560,  284.0337,  177.8439 | [M−H] ^−^ | CSE | NA |
| 51 | Hydroxymandelic acid | 3.60 | 168.0423 | 168.0423 | 0.00 | C_8_H_8_O_4_ | 167.0349,  123.0443,  93.0337 | [M−H] ^−^ | SCE | NA |
| 52 | *D*− (+) −Mannose | 26.09 | 180.0626 | 180.0634 | −4.44 | C_6_H_12_O_6_ | 131.6458,  113.3378,  89.2665 | [M−H] ^−^ | SPE | NA |
| 53 | *D*−2−Deoxyribose | 0.53 | 134.0573 | 134.0579 | 4.48 | C_5_H_10_O_4_ | 115.0029,  89.0234,  71.0128 | [M−H] ^−^ | SPE,  SCE,  CSE | NA |
| 54 | 3−Methoxytyrosine | 4.86 | 211.0853 | 211.0845 | −3.79 | C10H13NO4 | 124.0397,  94.0289 | [M−H] ^−^ | SPE,  SCE | NA |
| 55 | 3−Hydroxydecanedioic acid | 8.19 | 218.1139 | 218.1154 | 6.88 | C_10_H_18_O_5_ | 157.0866,  59.0128 | [M−H] ^−^ | SPE,  SCE,  CSE | NA |
| 56 | Cuscuta propenamide 1 | 10.96 | 313.1338 | 313.1314 | −7.66 | C_18_H_19_NO_4_ | 178.0509,  148.0526,  135.0444 | [M−H] ^−^ | SPE,  SCE,  CSE | NA |
| 57 | 4−Oxododecanedioic acid | 11.77 | 244.1308 | 244.1311 | −1.23 | C_12_H_20_O_5_ | 225.1133, 207.1028, 199.1344, 181.1299, 99.0079 | [M−H] ^−^ | SCE,  CSE | NA |
| 58 | Artesunate | 24.72 | 384.177 | 384.1784 | −3.64 | C_19_H_28_O_8_ | 321.3181,  167.7988,  87.4630 | [M−H] ^−^ | CSE | NA |
| 59 | Undecanedioic acid | 15.34 | 216.1361 | 216.1362 | −0.46 | C_11_H_20_O_4_ | 197.1181,  153.1280 | [M−H] ^−^ | SPE,  SCE,  CSE | NA |
| 60 | Tridecanedioic acid | 10.98 | 244.1687 | 244.1675 | 4.91 | C_13_H_24_O_4_ | 225.1133,  199.1338,  181.1227 | [M−H] ^−^ | SPE,  SCE, | NA |
| 61 | 2,5−Dihydroxybenzaldehyde | 7.14 | 138.0307 | 138.0317 | −7.24 | C_7_H_6_O_3_ | 108.0206,  92.9190 | [M−H] ^−^ | SPE,  SCE,  CSE | NA |

SPE (straw peels of ethyl acetate fraction), SCE (straw core of ethyl acetate fraction) and CSE (corn silk of ethyl acetate fraction), NA: relevant references were not identified in the literature search

Alam, R., Mahmood, R. A., Islam, S., Ardiati, F. C., Solihat, N. N., Alam, M. B., Lee, S. H., Yanto, D. H. Y. & Kim, S. 2023. Understanding the biodegradation pathways of azo dyes by immobilized white-rot fungus, Trametes hirsuta D7, using UPLC-PDA-FTICR MS supported by in silico simulations and toxicity assessment. *Chemosphere,* 313**,** 137505.

Alvarez-Fernandez, M. A., Cerezo, A. B., Canete-Rodriguez, A. M., Troncoso, A. M. & Garcia-Parrilla, M. C. 2015. Composition of nonanthocyanin polyphenols in alcoholic-fermented strawberry products using LC-MS (QTRAP), high-resolution MS (UHPLC-Orbitrap-MS), LC-DAD, and antioxidant activity. *J Agric Food Chem,* 63**,** 2041-51.

Bai, S., Li, P., Liu, J., Cui, C., Li, Q. & Bi, K. 2019. A UFLC-MS/MS method for the simultaneous determination of eight bioactive constituents from red wine and dealcoholized red wine in rat plasma: Application to a comparative pharmacokinetic study. *Biomed Chromatogr,* 33**,** e4437.

Balkrishna, A., Joshi, M., Tomer, M., Verma, S., Gujral, S., Mulay, V. P., Srivastava, J. & Varshney, A. 2022. Identification, Validation and Standardization of Bioactive Molecules Using UPLC/MS-QToF, UHPLC and HPTLC in Divya-Denguenil-Vati: A Penta-Herbal Formulation for Dengue Fever. *Chromatographia,* 85**,** 831-850.

Bashar, H. M. K., Juraimi, A. S., Ahmad-Hamdani, M. S., Uddin, M. K., Asib, N., Anwar, M. P., Rahaman, F., Karim, S. M. R., Haque, M. A., Berahim, Z., Nik Mustapha, N. A. & Hossain, A. 2022. Determination and Quantification of Phytochemicals from the Leaf Extract of Parthenium hysterophorus L. and Their Physio-Biochemical Responses to Several Crop and Weed Species. *Plants (Basel),* 11.

Bharwad, K. D., Shah, P. A., Shrivastav, P. S., Sharma, V. S. & Singhal, P. 2020. Quantification of fenoprofen in human plasma using UHPLC-tandem mass spectrometry for pharmacokinetic study in healthy subjects. *Biomed Chromatogr,* 34**,** e4708.

Bi, Q. R., Hou, J. J., Yang, M., Shen, Y., Qi, P., Feng, R. H., Dai, Z., Yan, B. P., Wang, J. W., Shi, X. J., Wu, W. Y. & Guo, D. A. 2017. A Strategy Combining Higher Energy C-Trap Dissociation with Neutral Loss- and Product Ion-Based MS(n) Acquisition for Global Profiling and Structure Annotation of Fatty Acids Conjugates. *J Am Soc Mass Spectrom,* 28**,** 443-451.

Cadiz-Gurrea, M. D., Fernandez-Arroyo, S., Joven, J. & Segura-Carretero, A. 2013. Comprehensive characterization by UHPLC-ESI-Q-TOF-MS from an <i>Eryngium bourgatii</i> extract and their antioxidant and anti-inflammatory activities. *Food Research International,* 50**,** 197-204.

Câmara Neto, J. F., Campelo, M. D. S., Cerqueira, G. S., De Miranda, J. a. L., Guedes, J. a. C., De Almeida, R. R., Soares, S. A., Gramosa, N. V., Zocolo, G. J., Vieira Í, G. P., Ricardo, N. & Ribeiro, M. 2022. Gastroprotective effect of hydroalcoholic extract from Agaricus blazei Murill against ethanol-induced gastric ulcer in mice. *J Ethnopharmacol,* 292**,** 115191.

Glaser, P., Dawid, C., Meister, S., Bader-Mittermaier, S., Schott, M., Eisner, P. & Hofmann, T. 2020. Molecularization of Bitter Off-Taste Compounds in Pea-Protein Isolates (Pisum sativum L.). *J Agric Food Chem,* 68**,** 10374-10387.

Jia, Y., Shen, J., Li, X., Xie, H., Wang, J., Luo, J., Wang, K. D., Liu, Q. & Kong, L. 2014. Identification and analysis of gastrodin and its five metabolites using ultra fast liquid chromatography electrospray ionization tandem mass spectrometry to investigate influence of multiple-dose and food. *J Chromatogr A,* 1358**,** 110-6.

Kajita, M., Niwa, T. & Watanabe, K. 1993. Analysis of urinary organic acids by liquid chromatography-atmospheric pressure chemical ionization mass spectrometry. *J Chromatogr,* 622**,** 263-8.

Kasiotis, K. M., Baira, E., Iosifidou, S., Manea-Karga, E., Tsipi, D., Gounari, S., Theologidis, I., Barmpouni, T., Danieli, P. P., Lazzari, F., Dipasquale, D., Petrarca, S., Shairra, S., Ghazala, N. A., Abd El-Wahed, A. A., El-Gamal, S. M. A. & Machera, K. 2023. Fingerprinting Chemical Markers in the Mediterranean Orange Blossom Honey: UHPLC-HRMS Metabolomics Study Integrating Melissopalynological Analysis, GC-MS and HPLC-PDA-ESI/MS. *Molecules,* 28.

Kodani, S. D., Bussberg, V., Narain, N. R., Kiebish, M. A. & Tseng, Y. H. 2022. Signaling Lipidomic Analysis of Thermogenic Adipocytes. *Methods Mol Biol,* 2448**,** 251-271.

Konczol, A., Engel, R., Szabo, K., Hornok, K., Toth, S., Beni, Z., Prechl, A., Mathe, I. & Tibor Balogh, G. 2014. Topical analgesic, anti-inflammatory and antioxidant properties of Oxybaphus nyctagineus: phytochemical characterization of active fractions. *J Ethnopharmacol,* 155**,** 776-84.

Lee, H. X., Li, W. M., Khatra, J., Xia, Z., Sannikov, O., Ling, Y., Zhu, H. & Lee, C. H. 2022. Antiproliferative Fatty Acids Isolated from the Polypore Fungus Onnia tomentosa. *J Fungi (Basel),* 8.

Li, J., Wang, Z., Fan, M., Hu, G. & Guo, M. 2022. Potential Antioxidative and Anti-Hyperuricemic Components Targeting Superoxide Dismutase and Xanthine Oxidase Explored from Polygonatum Sibiricum Red. *Antioxidants (Basel),* 11.

Li, S., Su, X., Abdullah, M., Sun, Y., Li, G., Cheng, X., Lin, Y., Cai, Y. & Jin, Q. 2018. Effects of Different Pollens on Primary Metabolism and Lignin Biosynthesis in Pear. *Int J Mol Sci,* 19.

Lin, Y. T., Mao, Y. W., Imtiyaz, Z., Chiou, W. F. & Lee, M. H. 2020. Comprehensive LC-MS/MS-based phytochemical perspectives and osteogenic effects of Uraria crinita. *Food Funct,* 11**,** 5420-5431.

Loo, Y. T., Howell, K., Suleria, H., Zhang, P., Gu, C. & Ng, K. 2022. Sugarcane polyphenol and fiber to affect production of short-chain fatty acids and microbiota composition using in vitro digestion and pig faecal fermentation model. *Food Chem,* 385**,** 132665.

Lu, X., Zheng, Y., Wen, F., Huang, W., Chen, X., Ruan, S., Gu, S., Hu, Y., Teng, Y. & Shu, P. 2021. Study of the active ingredients and mechanism of Sparganii rhizoma in gastric cancer based on HPLC-Q-TOF-MS/MS and network pharmacology. *Sci Rep,* 11**,** 1905.

Marchese, S., Curini, R., Gentili, A., Perret, D. & Rocca, L. M. 2004. Simultaneous determination of the urinary metabolites of benzene, toluene, xylene and styrene using high-performance liquid chromatography/hybrid quadrupole time-of-flight mass spectrometry. *Rapid Commun Mass Spectrom,* 18**,** 265-72.

Peixoto, J. a. B., Álvarez-Rivera, G., Alves, R. C., Costa, A. S. G., Machado, S., Cifuentes, A., Ibáñez, E. & Oliveira, M. 2021. Comprehensive Phenolic and Free Amino Acid Analysis of Rosemary Infusions: Influence on the Antioxidant Potential. *Antioxidants (Basel),* 10.

Popa, I., Solgadi, A., Pin, D., Watson, A. L., Haftek, M. & Portoukalian, J. 2021. The Linoleic Acid Content of the Stratum Corneum of Ichthyotic Golden Retriever Dogs Is Reduced as Compared to Healthy Dogs and a Significant Part Is Oxidized in Both Free and Esterified Forms. *Metabolites,* 11.

Pu, C. H., Lin, S. K., Chuang, W. C. & Shyu, T. H. 2018. Modified QuEChERS method for 24 plant growth regulators in grapes using LC-MS/MS. *J Food Drug Anal,* 26**,** 637-648.

Santos, P. S. M., Domingues, M. R. M. & Duarte, A. C. 2016. Fenton-like oxidation of small aromatic acids from biomass burning in atmospheric water and in the absence of light: Identification of intermediates and reaction pathways. *Chemosphere,* 154**,** 599-603.

Shen, S., Wang, J., Chen, X., Liu, T., Zhuo, Q. & Zhang, S. Q. 2019. Evaluation of cellular antioxidant components of honeys using UPLC-MS/MS and HPLC-FLD based on the quantitative composition-activity relationship. *Food Chem,* 293**,** 169-177.

Simayi, J., Abulizi, A., Nuermaimaiti, M., Khan, N., Hailati, S., Han, M., Talihati, Z., Abudurousuli, K., Maihemuti, N., Nuer, M., Zhou, W. & Wumaier, A. 2022. UHPLC-Q-TOF-MS/MS and Network Pharmacology Analysis to Reveal Quality Markers of Xinjiang Cydonia oblonga Mill. for Antiatherosclerosis. *Biomed Res Int,* 2022**,** 4176235.

Singh, A., Bajpai, V., Kumar, S., Sharma, K. R. & Kumara, B. 2016. Profiling of Gallic and Ellagic Acid Derivatives in Different Plant Parts of Terminalia arjuna by HPLC-ESI-QTOF-MS/MS. *Nat Prod Commun,* 11**,** 239-44.

Szewczyk, K., Miazga-Karska, M., Pietrzak, W., Komsta, L., Krzeminska, B. & Grzywa-Celinska, A. 2020. Phenolic Composition and Skin-Related Properties of the Aerial Parts Extract of Different Hemerocallis Cultivars. *Antioxidants (Basel),* 9.

Viacava, G. E., Roura, S. I., Lopez-Marquez, D. M., Berrueta, L. A., Gallo, B. & Alonso-Salces, R. M. 2018. Polyphenolic profile of butterhead lettuce cultivar by ultrahigh performance liquid chromatography coupled online to UV-visible spectrophotometry and quadrupole time-of-flight mass spectrometry. *Food Chem,* 260**,** 239-273.

Wroblewska, K. B., Plewa, S., Derezinski, P. & Muszalska-Kolos, I. 2019. Choline Salicylate Analysis: Chemical Stability and Degradation Product Identification. *Molecules,* 25.

Yan, Y., Fu, C., Cui, X., Pei, X., Li, A., Qin, X., Du, C. & Du, H. 2020. Metabolic profile and underlying antioxidant improvement of Ziziphi Spinosae Folium by human intestinal bacteria. *Food Chem,* 320**,** 126651.

Yang, L., Meng, X., Yu, X. & Kuang, H. 2017. Simultaneous determination of anemoside B4, phellodendrine, berberine, palmatine, obakunone, esculin, esculetin in rat plasma by UPLC-ESI-MS/MS and its application to a comparative pharmacokinetic study in normal and ulcerative colitis rats. *J Pharm Biomed Anal,* 134**,** 43-52.

Yin, N. W., Wang, S. X., Jia, L. D., Zhu, M. C., Yang, J., Zhou, B. J., Yin, J. M., Lu, K., Wang, R., Li, J. N. & Qu, C. M. 2019. Identification and Characterization of Major Constituents in Different-Colored Rapeseed Petals by UPLC-HESI-MS/MS. *J Agric Food Chem,* 67**,** 11053-11065.

Zeng, X., Su, W., Zheng, Y., Liu, H., Li, P., Zhang, W., Liang, Y., Bai, Y., Peng, W. & Yao, H. 2018. UFLC-Q-TOF-MS/MS-Based Screening and Identification of Flavonoids and Derived Metabolites in Human Urine after Oral Administration of Exocarpium Citri Grandis Extract. *Molecules,* 23.

Zhao, X., He, Y., Chen, J., Zhang, J., Chen, L., Wang, B., Wu, C. & Yuan, Y. 2021. Identification and direct determination of fatty acids profile in oleic acid by HPLC-CAD and MS-IT-TOF. *J Pharm Biomed Anal,* 204**,** 114238.

Zhou, Z., Deng, Z., Liang, S., Zou, X., Teng, Y., Wang, W. & Fu, L. 2023. Quantitative Analysis of Flavonoids in Fruiting Bodies of Sanghuangporus Using Ultra-High-Performance Liquid Chromatography Coupled with Triple Quadrupole Mass Spectrometry. *Molecules,* 28.
